# Selective Intratumoral Drug Release and Simultaneous Inhibition of Oxidative Stress by a Highly Reductive Nanosystem and Its Application as an Anti-tumor Agent

**DOI:** 10.7150/thno.38627

**Published:** 2020-01-01

**Authors:** Chunqi Zhu, Lihua Luo, Xindong jiang, Mengshi Jiang, Zhenyu Luo, Xiang Li, Weigen Qiu, Zhaolei Jin, Tianxiang Shen, Chunlong Li, Qingpo Li, Yunqing Qiu, Jian You

**Affiliations:** 1 College of Pharmaceutical Sciences, Zhejiang University, 866 Yuhangtang Road, Hangzhou, Zhejiang 310058, P. R. China;; 2 State Key Laboratory for Diagnosis and Treatment of Infectious Diseases, Collaborative Innovation Center for Diagnosis and Treatment of Infectious Diseases, The First Affiliated Hospital, Zhejiang University, 79 Qingchun Road, Hangzhou, Zhejiang 31003, P. R. China.

**Keywords:** Oxidative Stress, Docetaxel, Reductive nanosystem, Selective release, Systemic toxicity.

## Abstract

Excessive oxidative stress is always associated with the serious side effects of chemotherapy. In the current study, we developed a vitamin E based strongly reductive nanosystem to increase the loading efficiency of docetaxel (DTX, DTX-VNS), reduce its side toxicity and enhance the antitumor effect.

**Methods:** We used Förster Resonance Energy Transfer (FRET) to reveal the *in vivo* and *in vitro* fate of DTX-VNS over time. All FRET images were observed using the Maestro imaging system (CRI, Inc., Woburn, MA) and Fluo-View software (Olympus LX83-FV3000).

**Results:** Through FRET analyzing, we found that our nanosystem showed a selective rapider release of drugs in tumors compared to normal organs due to the higher levels of ROS in tumor cells than normal cells, and the accumulation of DTX at tumor sites in the DTX-VNS group was also notably more than that in the Taxotere group after 24 h injection. Meanwhile, DTX-VNS had a prominently stronger anti-tumor effect in various models than Taxotere, and had a synergistic effect of immunotherapy.

**Conclusions:** Our work presented a useful reference for clinical exploration of the *in vivo* behavior of nanocarriers (DTX-VNS), inhibition oxidative stress and selective release of drugs at tumor sites, thus reducing the side effects and enhancing the anti-tumor effects.

## Introduction

Unlike photodynamic therapy or radiotherapy, many chemotherapeutic agents have been demonstrated to have strong antitumor activity in the clinic, but not because of the toxicity of large amounts of reactive oxygen species (ROS) that are produced in cancer cells 1-4. However, ROS-independent chemotherapy still exhibits obvious toxic side effects during the treatment process, which seriously restricts its clinical application 5, 6. Most of the mechanisms of these side effects are related to excessive oxidative stress in cells 7, 8, that is attributed to an intercellular imbalance between pro-oxidant and antioxidant factors under the stimulation of the chemotherapeutic agents 9. ROS have been associated with the principle of oxidative stress-mediated pathological phenomena, which occur when there is a net increase in ROS, and this net increase has been implicated in cellular damage, inflammation and neurodegeneration 10-12. Moreover, much effort has been invested in defining the role of ROS as a tumor-promoting agent, with abundant evidence supporting this argument 13, 14.

Docetaxel (DTX), an antineoplastic agent, is a member of the second generation of the taxoid family derived from the needles of the European yew tree *Taxus baccata* 15. DTX is approximately twice as potent as paclitaxel in inhibiting microtubule depolymerization *in vitro* 16, 17. DTX has been used to treat a broad spectrum of solid tumors such as non-small cell lung cancer, locally advanced or metastatic breast cancer, and androgen-independent prostate cancer 18, 19. However, DTX also causes serious side effects, such as hemolytic toxicity, myelosuppression, anemia, and hypersensitivity reactions, which limit its clinical applications 20-22. Because of its poor solubility, DTX requires the addition of a substantial amount of surfactant (Tween) and ethanol in its commercial formulation (Taxotere, the only DTX preparation used in the clinic), which further increases the risk of systemic toxicity in cancer treatment 23.

As a major lipid soluble antioxidant present in the cell membrane, vitamin E plays an important role as a chain-breaking lipid antioxidant and free radical scavenger in the membrane of cells and subcellular organs 24-26. Vitamin E have been pursued as an anticancer agent because of its antioxidant properties, and it can also reduce ROS levels in tumor sites. Incomplete reduction of oxygen leads to the formation of the chemically more reactive species called ROS, including hydroxyl radicals, superoxide anions and other free radicals. Vitamin E mainly scavenges active free radicals by a hydrogen atom transfer reaction, producing non-free radical products and vitamin E radicals 27-29. Moreover, it has been reported that vitamin E may be a suitable candidate for the adjuvant treatment of cancer by antitumor immune regulation 30-32. It has been reported that vitamin E supplements can enhance the Th1-type immune response, while blocking the production of Th2 cytokines in different disease states 33, 34.

Currently, it is becoming a trend to alleviate chemotherapy by regulating the immune microenvironment of tumors and by similar nanodosing strategies 35, 36. Therefore, in this work, we expect to develop a highly reductive nanosystem loaded with DTX (DTX-VNS). Due to the regulated immune microenvironment, we hypothesized that the nanosystem could exhibit high antitumor efficacy, and synchronously reduce the imbalance of oxidative stress by eliminating the increased accumulation of ROS due to drug stimulation, resulting in decreased side effects during treatment. In addition, the* in vivo* fate of DTX-VNS over time after administration was revealed by Förster resonance energy transfer (FRET) analysis.Our nanosystem has a selective and more rapid release of drug in tumor sites than in normal organs because of the higher levels of ROS produced in tumors. The current research explored the *in vivo* behavior of reductive nanocarriers, which inhibited oxidative stress and selectively released drugs at tumor sites, and this may provide a useful reference for reducing the side effects and enhancing the efficacy of chemotherapeutic agents in the clinic.

## Materials and Methods

### Materials

Docetaxel (DTX) was purchased from Fujian Nanfang Pharmaceutical Co., Ltd. (Fujian, China); medium chain triglyceride (MCT), soybean lecithin (S100), vitamin E (VE, α-tocopheryloxyacetic acid), corn oil and soybean oil were purchased from Lipoid Co. (Ludwigshafen, Germany). Dulbecco's Modified Eagle Medium (DMEM), Roswell Park Memorial Institute (RPMI) 1640 medium, trypsin, fetal bovine serum (FBS) and penicillin/streptomycin (100 U/mL) were obtained from JiNuo Biotechnology Co., Ltd. (Zhejiang, China). 3-[4,5-Dimethylthiazol-2-yl]-2,5-diphenyltetrazolium bromide (MTT) and Hoechst33324 were acquired from Sigma-Aldrich Inc. (St Louis, MO, USA). EthD-1 and calcein AM (live/dead viability/cytotoxicity kit [L-3224]) were purchased from Life Technologies (Carlsbad, CA, USA). 3,3′-dioctadecyloxacarbocyanine perchlorate (DiO), 1,1'-dioctadecyl-3,3,3',3'-tetramethylindocarbocyanine perchlorate (DiI), 1,1'-dioctadecyl-3,3,3',3'-tetramethylindotricarbocyan ine Iodide (DiR), propidium iodide (DiD), and Nile Red (NR) were purchased from Invitrogen Co. (Carlsbad, CA, USA). Chemicals and solvents were of analytical grade and were used as received.

### Cell Culture and Animals

4T1 (mouse breast carcinoma), A549 (human pulmonary carcinoma), MDA-MB-231 (human breast carcinoma), LO2 (human hepatocytes), NIH-3T3 (mouse embryo fibroblast) and HEK 293 cell lines were purchased from the Institute of Biochemistry and Cell Biology (Shanghai, China). Cells were cultured at 37 °C in a humidified atmosphere containing 5% CO_2_ in RPMI 1640 medium or DMEM supplemented with 10% fetal bovine serum and 100 U/mL penicillin and 100 U/mL streptomycin.

All animal experiments were conducted in accordance with the National Institutes of Health Guide for the Care and Use of Laboratory Animals with the approval of the Scientific Investigation Board of Zhejiang University.

### Preparation and Characteristics of DTX-VNS

First, to obtain a nanosystem with high DTX encapsulation efficiency, the solubility of DTX in different oily materials was investigated. Briefly, excess DTX was added to distilled water (H_2_O), corn oil, soybean oil, MCT and VE, and then the mixtures were shaken at room temperature for 48 hours 37. The concentration of DTX in the medium was determined by high-performance liquid chromatography (HPLC) 38.

DTX-VNS were prepared by a high-energy emulsification method using high pressure homogenization (HPH). Briefly, 30 mg of DTX was dissolved in a mixed medium of VE, S100, and MCT (weight ratio of 2:1:1) to form an oil phase, which was further dispersed in an aqueous phase containing sucrose. DTX-VNS was finally obtained by dispersing the oil droplets into nanoscale-sized particles using the HPH method. DTX-NS (only DTX-loaded nanosystems, without VE) were prepared by the same method.

The mean droplet size and zeta potential of DTX-VNS were measured by dynamic light scattering (DLS) with a Zetasizer (ZS90, Malvern Co., UK). The morphology of DTX-VNS was observed by transmission electron microscope (TEM, JEOL JEM-1230 microscopy at 120 kV; JEOL, Japan). The encapsulation efficiency of DTX in the nanosystem was measured by ultrafiltration method.

### Antitumor Activity *in vivo*

The antitumor efficacy was assessed on 4T1, MDA-MB-231 and A549 tumor models. The 4T1 and MDA-MB-231 tumor models were obtained by orthotropic injection of 4T1 or MDA-MB-231 cells (1 × 10^6^) into the upper breast pad of each female BALB/c or nude mouse, respectively. A549 tumor models were established by subcutaneous injection of A549 cells (1 × 10^6^) into the right flank of each BALB/c mouse. Mice bearing different tumors were randomly divided into four or five groups (n=5 per group) when the tumor diameters reached 50-100 mm^3^. The mice in the different groups were intravenously injected with saline, blank nanosystem (Blank VNS, no DTX loading), DTX-VNS (10 mg DTX/kg per injection), DTX-NS (10 mg DTX/kg per injection) or Taxotere (10 mg DTX/kg per injection) for a total of 6 times (twice/per week), respectively. The tumor volumes and body weights of the mice were monitored. After treatment, the tumors and major organs were collected, weighed, sectioned for H&E (hematoxylin-eosin), Ki-67 or TUNEL staining.

### *In vitro* Synergistic Anticancer Effects

The synergistic anticancer effect of DTX and VE was first investigated using live & death cell staining. 4T1 cells were incubated with Blank VNS, DTX-VNS, Taxotere or Taxotere plus VE (a mixture of Taxotere and VE at 1:10, weight ratio, the same ratio of DTX/VE in DTX-VNS) at equivalent DTX concentrations of 10 μg/mL, 5 μg/mL and 1 μg/mL. After incubation for 48 h, the cells were stained with calcein AM and EthD-1 and then observed using a fluorescence microscope (Nikon, Japan).

4T1 cells were treated with DTX-VNS, Taxotere or Taxotere plus VE at the equivalent DTX concentration of 10 μg/mL for 24 or 48 hours. Apoptosis of the cells was detected by an Accuri^TM^ C6 flow cytometer (FC500 MCL; Beckman Coulter, Miami, FL) after staining with an annexin V-fluorescein isothiocyanate apoptosis detection kit I (MultiSciences Biotech, Zhejiang, China) according to the manufacturer's instructions.

For the cell cycle distribution study, 4T1 cells were treated with Blank-VNS, DTX-VNS, Taxotere or Taxotere plus VE at the equivalent DTX concentration of 0.5 μg/mL for 48 hours. The phase of cell cycle arrest was evaluated by flow cytometry using propidium iodide as a fluorescent marker 17, 39.

### Synergistic Effects from Immunotherapy

The mice bearing 4T1 tumors were divided into 5 groups (5 mice per group). The mice in groups 1-5 were intravenously injected with saline, Blank-VNS (100 mg VE/kg), Taxotere (10 mg DTX/kg), DTX-VNS (10 mg DTX/kg and 100 mg VE/kg), and Taxotere (10 mg DTX/kg) plus VE (100 mg/kg) three timesat a frequency of once a week. After the treatments for three weeks, the mice were sacrificed, and the tumors, spleens and lymph nodes were collected. Fresh tumor tissues were sliced to analyze CD8+ T cells and IFN-γ levels using immunofluorescence. T cells (CD3+, CD4+, and CD8+) in the spleen and DC cells in lymph nodes were isolated and analyzed using flow cytometry, and IL-4, IL-2 and IFN-γ in tumor and spleen tissues were also examined using ELISA kits.

### Bone Marrow Toxicity and Hemolysis Test

Myelosuppression toxicity is one of the most common side effects of DTX. To investigate the myelosuppression toxicity of DTX-VNS, two-week-old SD rats were sacrificed, and the bone marrow cells were isolated from the tibias. Then the cells (5 × 10^3^ cells/well in MethoCult medium) were treated with Blank-VNS, DTX-VNS, Taxotere or Taxotere plus VE at equivalent DTX concentrations of 0.1, 1 and 10 μg/mL. Mock-treated cells were used as the control (Ctrl). Cells were incubated at 37 °C and 5% CO_2_ for about approximately 14 days until colonies formed and the numbers were counted under the observation of microscope after crystal violet staining.

For the hemolysis test, fresh blood was collected from a healthy rabbit's heart. The blood was diluted with saline (1:10, v/v) and centrifuged at 1300 rpm for 15 min to separate the red blood cells. The cells in saline were further incubated with Blank-VN, DTX-VNS, Taxotere, Taxotere plus VE, or the DTX-loaded nanosystem without VE (DTX-NS) at various DTX concentrations. The cells treated with only pure water and saline were used as positive and negative controls, respectively. After 3 hours of incubation at 37 °C, the samples were centrifuged at 3000 rpm for 5 min and the supernatants were carefully collected for spectroscopic analysis at 545 nm using an ultraviolet spectrophotometer (UNIC-7200, China). The hemolysis parameters were calculated following the formula (OD_t_-OD_n_)/(OD_p_-OD_n_) × 100%, where OD_t,_ OD_n_ and OD_p_ represent the optical densities of the test, negative and positive groups, respectively 40, 41.

### Selective Drug Release *in vitro*

*In vitro* DTX release assays were performed in media with varying concentrations of H_2_O_2_ (a mixture of SDS and ethanol at a weight ratio of 1:5) using dialysis bags (MWCO 3500 Da). An aqueous dispersion of DTX-VNS (1 mg in 0.5 mL) was placed into the dialysis bags which were then placed in excessive medium (40 mL) and incubated at 37°C with different concentrations of H_2_O_2_ (1 μm, 0.1, 1, and 10 mM). The release medium was collected at predetermined intervals and replaced with an equal volume of fresh medium. The release of DTX in DTX-NS was performed at a concentration of 0.1 mM H_2_O_2_.The DTX content in the release medium was measured by HPLC analysis.

### Intracellular ROS Determination

To detect ROS accumulation in cells under different treatments, 4T1 tumor or LO2 normal cells were incubated with Blank-VNS, DTX-VNS, Taxotere or Taxotere plus VE at the equivalent DTX concentration of 10 μg/mL for 48 hours. Cells with no treatment were used as controls (Ctrl). The cells were then treated with DCFH-DA (10 μM) for 20 min to detect intracellular ROS. Then, the fluorescence of the samples was examined with a fluorescence microscope.

### *In vivo* Acute Toxicity

For the acute toxicity study, SD rats (200-225 g) were divided into 6 groups (7 rats/group). The rats in groups 1-6 were intravenously injected with saline, Blank-VNS, DTX-VNS (low dose, 8 mg DTX/kg), DTX-VNS (high dose, 16 mg DTX/kg), Taxotere (low dose, 8 mg DTX/kg), and Taxotere (high dose, 16 mg DTX/kg) four times (once per week). The rats were weighed and visual observations of mortality, behavioral patterns, changes in physical appearance and signs of illness were conducted once daily during the period. At the end of the experiments, blood was collected for biochemical and hematological analyses. Then, the rats were sacrificed, and the organs were weighed to calculate the organ coefficient.

### Tolerance Dose Study

BALB/c mice were divided into 2 groups (3 mice/group), and were intravenously injected with DTX-VNS (20 mg DTX/kg per injection) or Taxotere (20 mg DTX/kg per injection) for four weeks (two injections per week). Animal mortality was recorded during the experiment. Then the animals continued to be reared normally, and their recovery was assessed by visual observation.

### Selective Drug Release and Toxicity

For the selective drug release study, DiO and Dil (1:5 w/w) were co-encapsulated into DTX-VNS. Cancer (4T1, MDA-MB-231 and A549) and normal cells (LO2, HEK293 and NIH-3T3) were incubated with DiO and Dil labeled DTX-VNS for different time. FRET images were obtained using a fluorescence microscope (480 nm excitation, 490-520 nm emission for DiO and 560-650 nm for DiI). All images were obtained and processed with Fluoview software (Olympus LX83-FV3000). The fluorescence intensity of the disintegrated and integrated DTX-VNS in cells was calculated using ImageJ software.

Selective drug release was further investigated in mice bearing various tumors (4T1, MDA-MB-231 and A549) after intravenous injection of DTX-VNS, which was colabeled with NR and DiD. At the predetermined time point, mice were anesthetized and FRET images were observed using the Maestro imaging system (CRI, Inc., Woburn, MA) (590 nm excitation, 600-630 nm emission for NR and 670-700 nm emission for DiD). Mice were then sacrificed, and the main organs including tumors were collected and further imaged. The fluorescence intensities of the disintegrated and integrated DTX-VNS in various organs 24 hours after injection were calculated using ImageJ. To investigate the role of VE in the selective release of DTX-VNS, A549-bearing mice were injected with NR or DiD-loaded DTX-NS (no VE), and the fluorescence images were acquired.

The toxicity of DTX-VNS to cancer and normal cells was determined by MTT assay according to the manufacturer's suggested procedures. A549, 4T1, MDA-MB-231, LO2, NIH 3T3 and HEK 293 cells were exposed to DTX-VNS or Taxotere for 48 hours. The data are expressed as the percent of surviving cells and are reported as the mean values of 5 measurements.

### Biodistribution Study

A 4T1 tumor model, generated by the subcutaneous injection of 1 × xX10^6^ cells in 100 μL of PBS into the right rear flanks of male BALB/c mice (aged 6-8 weeks), was employed for imaging. When the tumors reached approximately 200 mm^3^ in size, the mice bearing 4T1 tumors were injected via the tail vein with DiR-loaded VNS. The mice were optically imaged using an *in vivo* imaging system (Cambridge Research & Instrumentation, Inc., Woburn, MA) at predetermined times after the injection. At the end of the experiment, the mice were sacrificed. Various tissues, including the tumors were collected, weighed, and observed by the *in vivo* imaging system, and the fluorescence intensity of DiR was also obtained. The accumulation of DiR in various tissues was calculated as the signal-to-weight ratio and expressed as the mean ± SD.

Mice bearing MDA-MB-231 tumors were divided into 2 groups (6 mice/group) and intravenously injected with DTX-VNS or Taxotere at a DTX dose of 10 mg/kg. At 24 hours postinjection, the mice were sacrificed, and the main organs including the tumors, were collected and homogenized. The DTX content in various organs was analyzed by HPLC-MS after DTX extraction from the homogenate 42.

### Statistics

Statistically significant differences between multiple values were determined with ANOVA, followed by Tukey-Kramer tests. Differences between pairs of groups were analyzed by Student's t-test, and P-values < 0.05 were considered statistically significant.

## Results

### Preparation and Characterization of DTX-VNS

**Figure 1:**
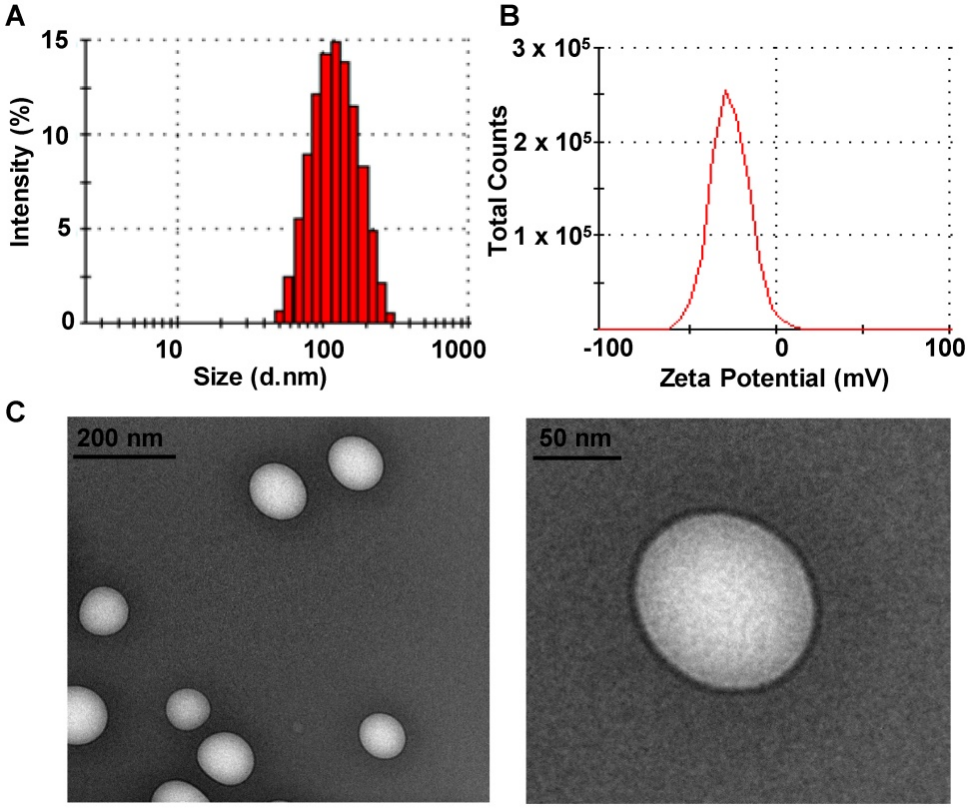
** Characteristics of DTX-VNS.** Size distribution **(A)** and Zeta Potential **(B)** of DTX-VNS. **(C)** TEM images of DTX-VNS.

The solubility of DTX in different media at room temperature was investigated. Among the media tested, DTX presented the highest solubility in VE, which would facilitate the efficient encapsulation of DTX into VE-based nanosystems (data not shown) 37, and DTX-VNS demonstrated a high encapsulation efficiency for DTX (97.45 ± 0.96%). Our DTX-VNS had a mean diameter of 107.5 ±1.8nm, as determined by DLS (Figure 1A), as well as a negative surface charge (-37.1± 1.2 mV) (Figure 1B). TEM showed complete spherical morphology and a smooth DTX-VNS surface of (Figure 1C). The stability of the DTX-VNS is presented at Table S1.

### *In vivo* Anticancer Activity

**Figure 2:**
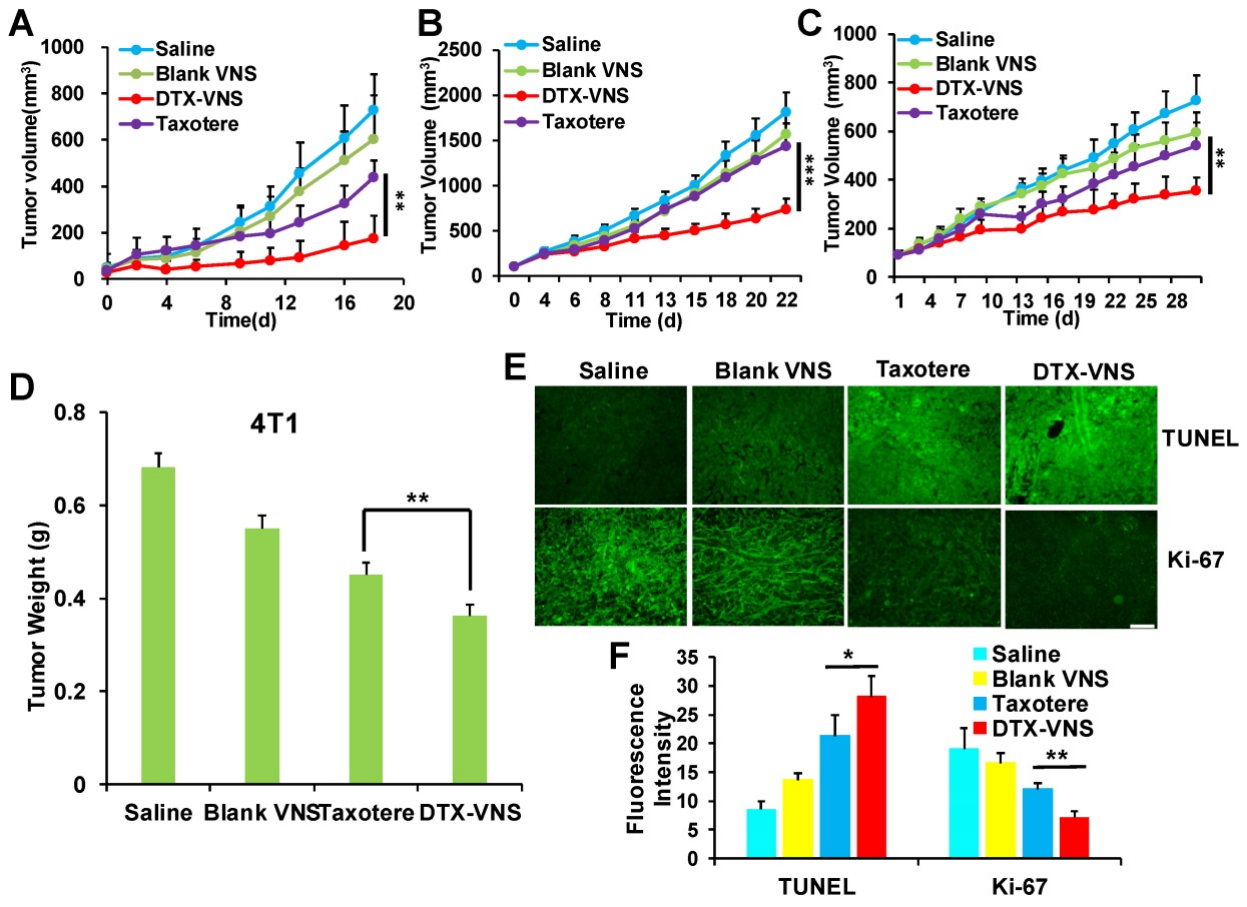
***In vivo* anticancer activity.** Tumors growth curves for the mice bearing 4T1 tumors **(A)**, MDA-MB-231 tumors **(B)** and A549 tumors **(C)** after treatment. *p < 0.05, **p < 0.01, ***p <0.005. **(D)** Average tumor weight of each group of 4T1 tumor model at the end of the study. Representative TUNEL and Ki-67 staining images **(E)** and quantitative analysis **(F)** of 4T1 tumor tissues for four groups; the scale bar is 100 μm.

The antitumor activity of DTX-VNS was tested in 4T1, MDA-MB-231, and A549 tumor models. Surprisingly, DTX-VNS showed a significantly stronger ability than Taxotere to inhibit the growth of three kinds of tumors under the same DTX dose (Figure 2A-C). At the end of the experiment, the tumors from each group were collected. The average tumor weight in the DTX-VNS group was the smallest among the groups, further demonstrating the stronger antitumor efficacy of our nanosystem (Figure 2D, S1). The 4T1 tumor in the DTX-VNS group showed more serious cancer cell apoptosis and a significant decrease in proliferative activity (Figure 2E-F). In addition, normal tissues did not show significant damage or inflammation (Figure S2A), and there was no significant loss in body weight throughout the experiment (Figure S2B), which suggested low systemic toxicity of DTX-VNS. To explicitly exclude the possible effects of the nanovehicles, we also used DTX-NS as a control. From the results in figure S3, we can see that the anti-tumor effects of nanoparticulate DTX (DTX-NS) and Taxotere were essentially the same and were not as good as those observed for DTX-VNS. Our nanosystem exhibited significantly enhanced anti-tumor activity, driving a series of studies further implemented to reveal the possible mechanism.

### Cancer Cell Killing Activity

**Figure 3:**
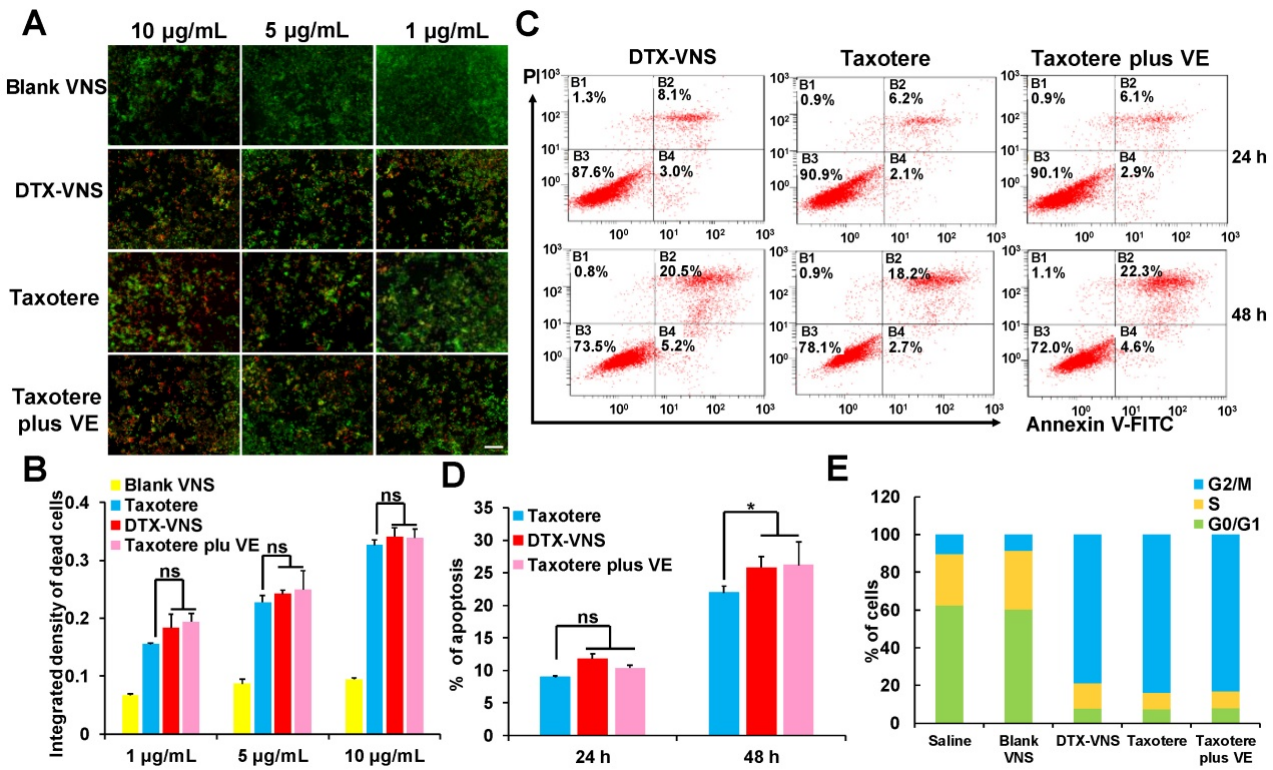
***In vitro* anticancer activity.** Imaging **(A)** and quantitative analysis **(B)** based on the imaging of the dead (red) and live (green) cell assay with exposure of the 4T1 cells to different dose of Blank VNS, DTX-VNS, Taxotere, and Taxotere plus VE; the scale bar is 100 μm. Dot plot representing level **(C)** and quantitative analysis **(D)** of apoptosis after treating 4T1 cells with DTX-VNS, Taxotere, and Taxotere plus VE at equivalent drug concentration 10 μM for 24 hours (up) and 48 hours (down). *p < 0.05, **p < 0.01, ***p <0.005. **(E)** Cell cycle phase distribution of 4T1 cells after treatment for 48 h, the concentration of DTX was 0.5 μg/mL.

Compared to Taxotere, DTX-VNS exhibited enhanced cancer cell killing effects under a cell incubation concentration of 1-10 μg/mL, as analyzed by live & death cell staining. This result should be attributed to the fact that VE was contained in the nanosystem, because the killing activity of Taxotere also slightly increased when it was combined with VE (Figure 3A-B). These results were further confirmed by a cell apoptosis assay using flow cytometry after the double staining of PI and annexin V, which showed that VE causes a small increase in apoptosis (Figure 3C-D). Cell cycle profiles were also analyzed by flow cytometry after propidium iodide staining. Treatments with DTX-VNS, Taxotere and Taxotere plus VE for 48 hours caused similar cell cycle arrest in the G2-M phase, which presented a DTX antitumor mechanism of inhibiting microtubule depolymerization and destroying the mitosis of cancer cells. Treatment with Blank-VNS did not exhibit similar anti-cancer characteristics and was similar to saline treatment (Figure 3E). These results indicated that although VE could help DTX exert a stronger cancer cell killing effect, it did not contribute to the improvement of the direct lethality.

### Synergistic Immunotherapy Effect

**Figure 4:**
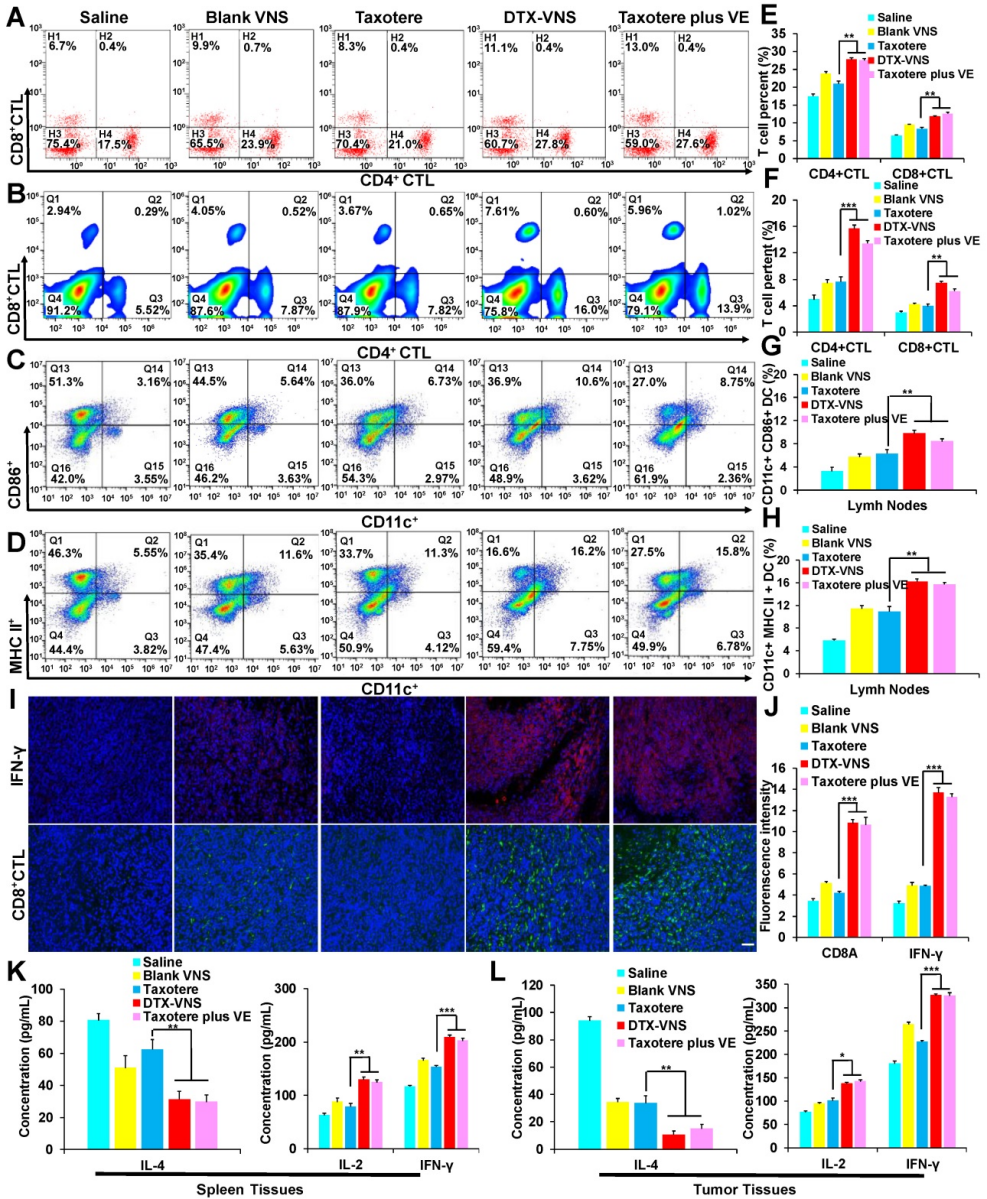
***In vivo* synergetic immunotherapy effects. (A)** Representative flow cytometry plots showing different groups of T cells in splenic lymphocytes. Representative flow cytometry plots showing **(B)** CD4^+^, CD8^+^ T cells, **(C)** CD86^+^ DC cells and **(D)** MHC II^+^ DC cells in lymph node lymphocytes. **(E-H)** Quantitation based on the results in panel A-D. **(I)** Immunoflorescence was used to examine IFN-γ (upper) and CD8+ CTL (lower) in tumor sections at the end of treated with Saline, Blank VNS, DTX-VNS, Taxotere, and Taxotere plus VE (red IFN-γ-positive cells; green, CD8^+^ CTL-positive cells; blue, cell nuclei); the scale bar is 100 μm. (**J**) The fluorescence intensity of CD8^+^ CTL and IFN-γ in tumors was calculated based on **(I)** using ImageJ. IL-4, IL-2, and IFN-γ levels in the splenic tissues **(K)** and tumor tissues **(L)** of each group of mice isolated at the end of different treatments detected using ELISA. *p < 0.05, **p < 0.01, ***p <0.005. The error represents the standard deviation from the mean (n = 5).

Vitamin E has been reported to exhibit an antitumor immune effect, for example by enhancing the Th1-type immune response and hindering Th2 cytokine production 43, 44. The numbers of CD3, CD8 and CD4-positive T cells in the spleen and CD11c-, CD86- and MHC II- positive DC cells in the lymph nodes were measured and presented stronger proliferation in the DTX-VNS and Taxotere plus VE groups than in the other groups (Figure 4A-D, E-H). The mice bearing 4T1 tumors were sacrificed after various treatments, and the tumors were sliced for the analysis of IFN-γ levels and the infiltration of CD8-positive cytotoxic T lymphocytes (CD8^+^CTL). VE alone (Blank-VNS group) or DTX alone (Taxotere group) caused only a slight increase in IFN-γ levels and CD8^+^CTL infiltration in the tumors, compared with the saline group. Interestingly, the levels of both IFN-γ and CD8^+^CTL were significantly improved in the tumors after treatment with a combination of DTX and VE, as presented in the DTX-VNS and Taxotere plus VE groups (Figure 4I-J). Furthermore, the immune responses of Th1 and Th2 were further monitored in the tumor and spleen using ELISA after treatments. The Th1 immune response was significantly upregulated through an increase in the secretion of IL-2 and IFN-γ, while the Th2 response was obviously downregulated, as evidenced by a significant reduction in IL-4 secretion in the DTX-VNS and Taxotere plus VE groups (Figure 4K-L). The results indicated that, compared to VE or DTX alone, VE with the help of DTX chemotherapy could more efficiently enhance the immune response that occurs in the tumor microenvironment, which may be a main reason why DTX-VNS exhibited significantly enhanced anti-tumor activity.

### Myelosuppression and Hemolytic Toxicity

**Figure 5:**
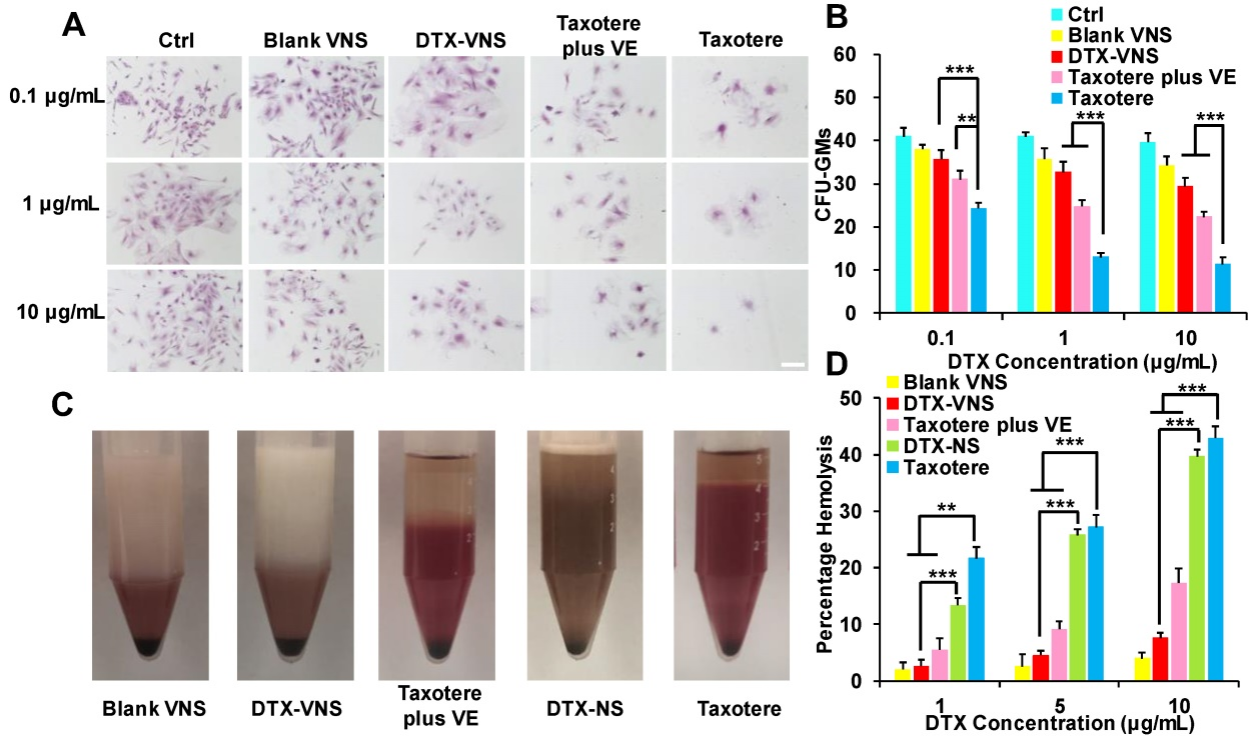
** The detoxification of DTX-VNS. (A)** Bone marrow cells from mice were incubated Blank VNS, DTX-VNS, Taxotere, and Taxotere plus VE at the dose of 0.1 μg/mL, 1 μg/mL or 10 μg/mL for 14 days in methylcellulose-based media. Mock-treated cells were used as the control; the scale bar is 50 μm. **(B)** CFU-GMs Colonies of colony-forming units that generate granulocytes and macrophages were counted using an inverted microscope. **(C)** Photographs of defibrinated blood mixed with Blank VNS, DTX-VNS, Taxotere plus VE, DTX-NS, and Taxotere in middle concentration (5 μg/mL) for 3 hours. **(D)** Percentage hemolysis observed after incubating different tests with RBCs up to 3 hours. The date are presented as mean ± standard error from three experiments. *p < 0.05, **p < 0.01, ***p <0.005.

Myelosuppression is a common side effect caused by clinical chemotherapy. Primary mouse bone marrow cells were isolated and treated with Blank-VNS, DTX-VNS, Taxotere or Taxotere plus VE. Then, the CFUs were counted under a microscope, including CFU-GMs (the colonies that contained 30 to thousands of granulocytes, macrophages, or both cell types), erythroid progenitors (BFU-Es, made up of erythroid clusters and a minimum of 30 cells), and CFU-GEMMs (which tend to produce large colonies of 500 cells containing erythroblasts and recognizable cells of at least two other lineages). Taxotere caused a significant decrease in the amount of CFU-GMs, BFU-Es and CFU-GEMMs after incubation with a concentration of 0.1-10 μg/mL DTX. However, in the same concentration range, the degree of decline of the CFUs after the treatment with of DTX-VNS or Taxotere plus VE was greatly reduced (Figure 5A-B and S4A-C). These results indicated that, unlike Taxotere alone, DTX-VNS could efficiently alleviate the myelosuppressive toxicity caused by DTX, which should be attributed to the role of VE in the nanosystems.

The hemolytic potential of DTX-VNS was assessed using fresh RBCs in DTX concentration range of 1-10 μg/mL. In the low DTX concentration range of 1-5 μg/mL, similar to Blank-VNS, DTX-VNS did not cause significant hemolytic toxicity. Even at the highest DTX concentration of 10 μg/mL, only 7.53% hemolysis occurred. However, at the same concentration of 10 μg/mL, DTX-NS (no VE) and Taxotere alone exhibited obviously increased levels of hemolysis (39.69 ± 1.14% and 42.89 ± 2.05%, respectively) (Figure 5C-D and S4D). These data demonstrated that DTX-VNS had significantly reduced hemotoxicity compared to Taxotere.

### Selective Drug Release *in vitro*

**Figure 6:**
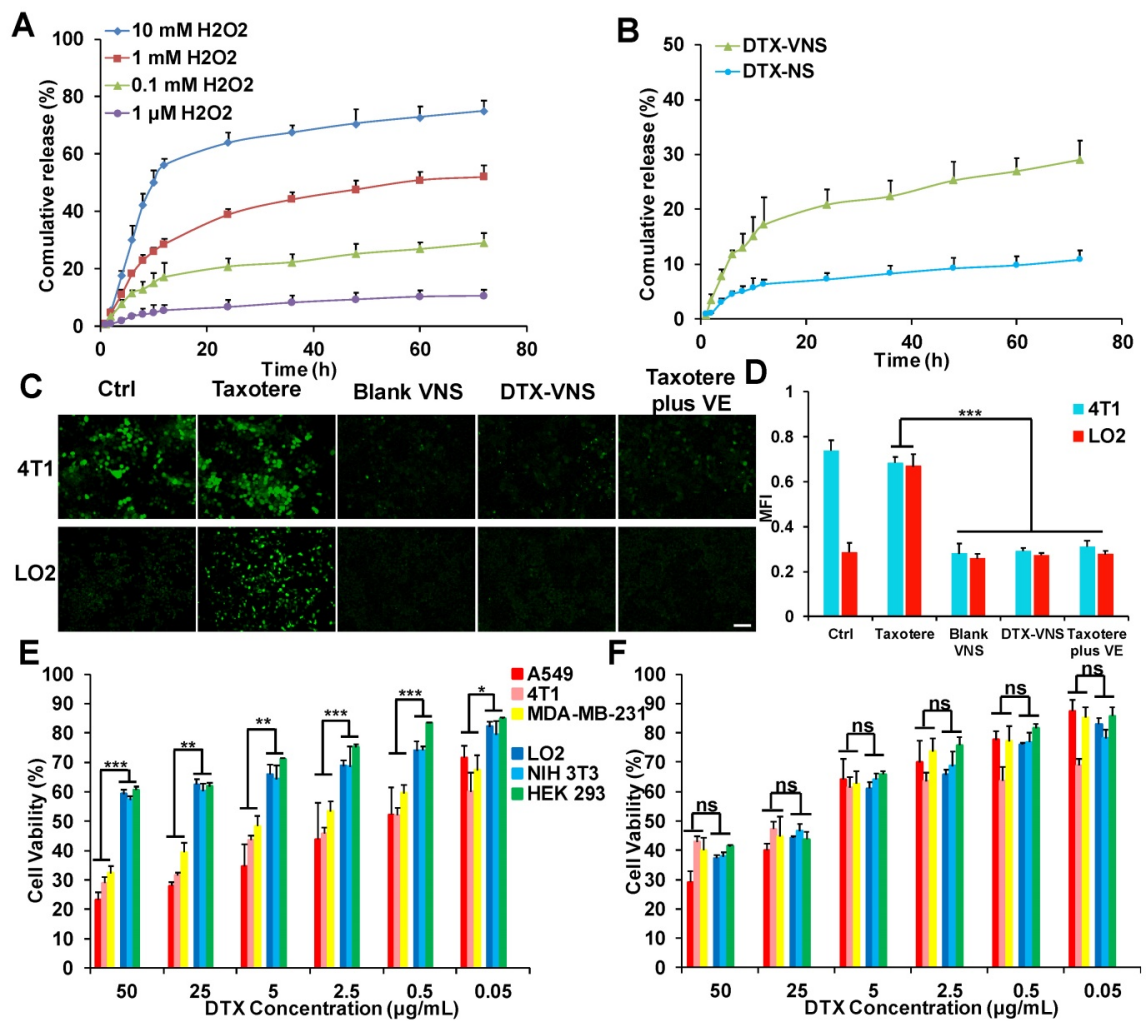
** ROS-responsive drug release and selective toxicity *in vitro*. (A)** *In vitro* DTX release from DTX-VNS against different level of H_2_O_2_ at 37 °C. **(B)** *In vitro* DTX release from DTX-VNS and DTX-NS, respectively. The concentration of H_2_O_2_ was 0.1 mM. Fluorescence microscopy images **(C)** and corresponding fluorescence intensities **(D)** of intracellular ROS level in 4T1 tumor cells and LO2 normal cells treated with Taxotere, Blank VNS, DTX-VNS, and Taxotere plus VE for 48 hours. Scale bars, 100 μm. MTT assay of three tumor cells (A549, 4T1, MDA-MB-231) and three normal cells (LO2, NIH 3T3, HEK 293) treated with DTX-VNS **(E)** or Taxotere **(F)** for 48 hours (n=5). All error bars are expressed as ± SD, n=5; *p < 0.05, **p < 0.01, ***p <0.005.

From the* in vitro* drug release studies, we can see that when the concentration of H_2_O_2_ is 0.1 μM (basically equivalent to physiological conditions), only 10% DTX was released. However, when the concentration of H2O2 was 0.1 mM (similar to the level of ROS in cancer cells), nearly 29% of DTX was released. When the concentration of ROS further increased to 1 mM, 10 mM, the release of DTX increased to 52%, 75%, respectively (Figure 6A). However, in figure 6B, only 10% of DTX was released from DTX-NS even at tumor cell ROS levels (0.1 mM). These results indicated that the ROS-responsive VE nanoparticles (DTX-VNS) can release a small amount of DTX in cancer cells, and higher levels of ROS can trigger a greater DTX release.

### Intercellular ROS Determination

Excessive oxidative stress is frequently associated with the mechanism of systemic toxicity caused by chemotherapy 45, and the accumulation of intracellular ROS is a major factor in oxidative stress. DTX-VNS demonstrated the ability to significantly down regulate ROS levels in cancer cells. Blank-VNS and Taxotere plus VE also exhibited similar effects. In normal cells, incubation with Taxotere resulted in a significant increase in intracellular ROS, suggesting a potential upregulated of oxidative stress of the cells, whereas the cells maintained a low intracellular ROS level before and after treatment with DTX-VNS, which was due to the antioxidant effects of VE (Figure 6C-D). DTX-VNS has significantly reduced toxic side effects, which is most likely achieved by consuming ROS and alleviating the intercellular oxidative stress level.

### *In vivo* Acute Toxicity and Tolerance Dose

An *in vivo* acute toxicity test was performed in rats to further verify the systemic toxicity of DTX-VNS after four intravenous injections with a total DTX dose of 32 or 64 mg/kg. The main hematology (Table S2) and clinical chemistry parameters (Table S3) did not show significant differences between the DTX-VNS, Blank VNS and Saline groups at the low and high treatment doses. However, some pathological indexes such as MONO and CK values, in the Taxotere group showed obvious abnormalities compared with the saline group. Furthermore, no obvious body weight loss (Figure S5A) and relative organ weight changes of the animals (Figure S5B) were found in the DTX-VNS group. These results strongly suggested that DTX-VNS had a low systemic toxicity, even at the injection doses of up to 64 mg/kg.

After four weeks of treatment (total 160 mg DTX/kg), all mice in the DTX-VNS group survived. However, in the Taxotere group, mouse deaths occurred during the second and third weeks. In the second week of administration (total 80 mg DTX/kg), the hind limbs of the mice in the Taxotere group showed slight stiffness, which became more serious with continued treatment. In the DTX-VNS group, slight stiffness occurred only after the third week of administration (total 120 mg DTX/kg) (Figure S6). These results suggested the significantly reduced neurotoxicity of DTX-VNS compared with Taxotere. Within one week after stopping treatment, the stiffness was significantly alleviated and the weight of the mice returned to normal levels. Compared with Taxotere, the animals showed significantly higher dose tolerance to DTX-VNS, which should be mainly due to the lower systemic toxicity of the nanosystem.

### Selective Toxicity in Normal and Tumor Cells

The killing effects of DTX-VNS and Taxotere against various tumor cells (A549, 4T1, MDA-MB-231) and normal cells (LO2, NIH 3T3, HEK 293) were investigated using a MTT assay. Taxotere showed similar lethality to normal and tumor cells. Interestingly, significantly more tumor cells were killed and more normal cells could survive under the same incubation conditions as DTX-VNS. For example, DTX-VNS caused nearly 70% of the tumor cells to be killed, while almost 60% of the normal cells survived at 50 μg DTX/mL incubation concentration (Figure 6E). After Taxotere treatment at the same concentration, 60-70% of cancer cells and normal cells died (Figure 6F). We also tested the killing effects of DTX-NS, which were similar to Taxotere, had the same killing effects on normal cells and tumor cellswith no selective killing effect (Figure S7). These results indicated that DTX-VNS could exhibit selective toxicity between normal and tumor cells.

### Selective Drug Release in Normal and Tumor Cell

**Figure 7:**
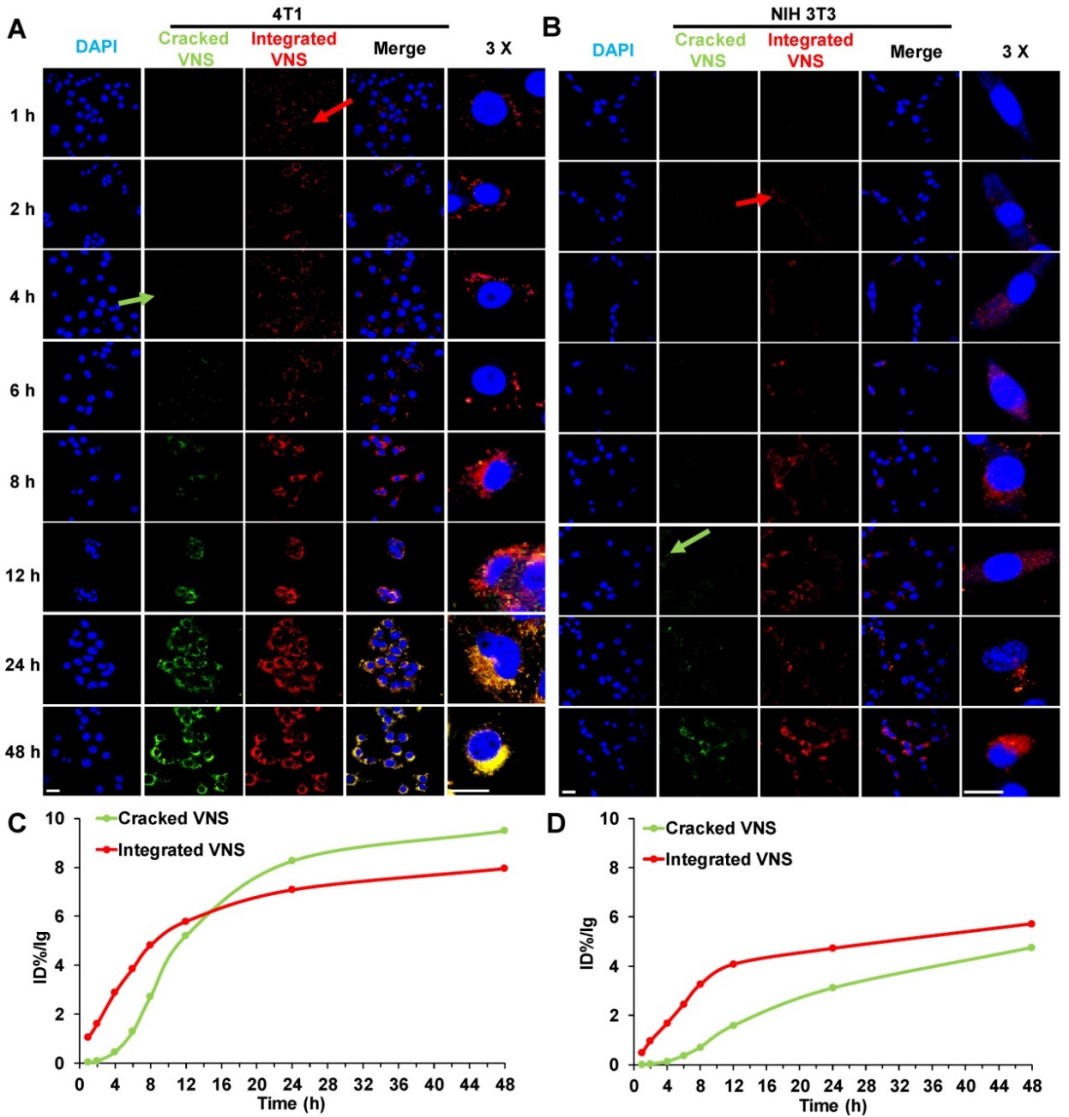
***In vitro* selective release of DTX-VNS.** Cellular uptake of DiO-, DiI-loaded DTX-VNS were incubated with 4T1 tumor cells **(A)** and NIH 3T3 normal cells **(B)** at 37 °C for predetermined times (blue: cell nuclei; green: Cracked VNS; red: Integrated VNS), and the fluorescence images inspected by confocal fluorescence microscopy. The fluorescence intensity of Cracked VNS and Integrated VNS in 4T1 tumor cells **(C)** or NIH 3T3 normal cells **(D) was** calculated based on **(A)** or** (B)** using ImageJ; the scale bar is 20 μm.

To explain the mechanism of DTX-VNS selective toxicity, we further investigated the drug release behavior of the nanosystem in normal and tumor cells. DTX-VNS with FRET effects with 480-nm excitation was first obtained by coencapsulating DiO and DiI into the nanosystem (Figure S8). DiO or Dil release behavior from DTX-VNS could be monitored by analyzing the fluorescence intensity at 575 nm and 505 nm. It was found that DTX-VNS could rapidly enter 4T1 tumor cells after incubation for one hour by the observation of red fluorescence (Integrated VNS, red arrow). After 4 hours of incubation, DiO or Dil began to be released from DTX-VNS because of the appearance of green fluorescence (Cracked VNS, green arrow) (Figure 7A). The uptake of DTX-VNS by normal cells was significantly lower than that of tumor cells at the same time point. Red fluorescence in NIH3T3 normal cells was observed for 2-4 hours after incubation (red arrow), but green fluorescence was not observed until 12 hours later (green arrow) (Figure 7B). Similar results were observed in the other tumor cells (A549 and MD-MBA-231) (Figure S9) and normal cells (LO2 and HEK293) (Figure S10). Semiquantitative results of the fluorescence intensity further confirmed that the signal in the tumor cells induced by the released DiO or Dil will gradually be stronger than that generated by the encapsulated fluorescence agents with the extension of incubation time (Figure 7C). However, the intensity of green fluorescence in normal cells was always significantly lower than that of the red fluorescence throughout the incubation period (Figure 7D). These data indicated the selective cellular internalization and drug release behavior of DTX-VNS, which may be responsible for the greater killing activity of the nanosystem to cancer cells.

### Selective Drug Release *in vivo*

**Figure 8:**
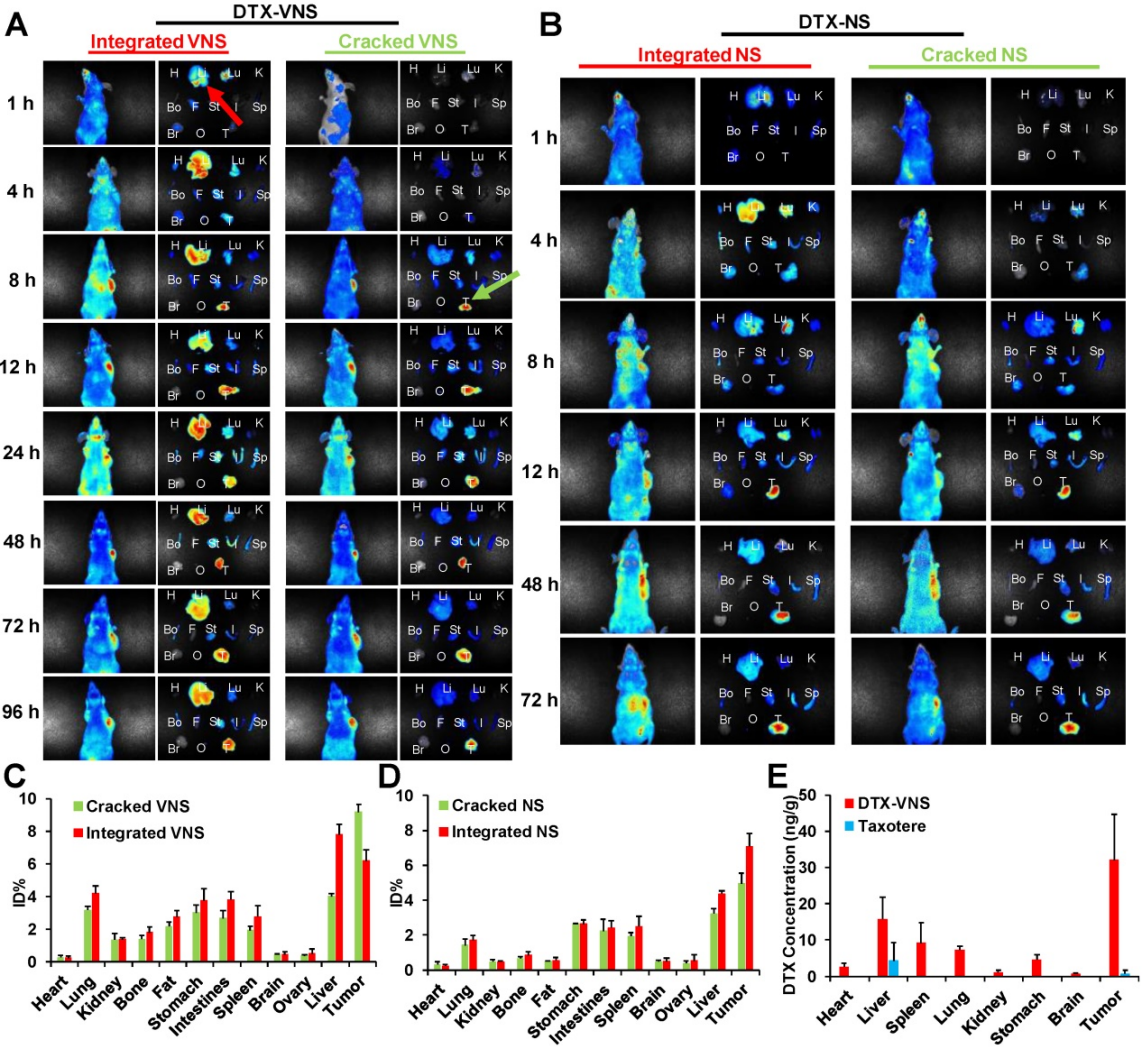
***In vivo* and ex *vivo* selective release of DTX-VNS.** *In vivo* and *ex vivo* images of the mice bearing A549 tumors and various tissues (H: heart, Li: liver, Lu: lung, K: kidney, Bo: bone, F: fat, St: stomach, I: intestines, Sp: spleen, Br: brain, O: ovary, and T: tumor) were acquired at predetermined times post i.v. injection of DTX-VNS **(A)** or DTX-NS **(B)**. %ID/g (percentage of the injected dose per gram of tissue) was analyzed to show the accumulation of cracked VNS (NS) and integrated VNS (NS) in various tissues at 48 hours after i.v. injection of DTX-VNS **(C)** or DTX-NS **(D)**. **(E)** The DTX concentration in various tissues (heart, liver, spleen, lung, kidney, stomach, brain and MDA-MB-231 tumor) at 24 hours after i.v. injection of DTX-VNS and Taxotere (n=5).

The selective drug release behavior was further investigated in mice bearing tumors after an intravenous injection of DTX-VNS with NR and DiD cocapsulation, which could present a FRET effect under an excitation of 590-nm and emission peaks of 618- and 667-nm, as the signals of the released and encapsulated fluorescence agents, respectively (Figure S11). The emission signal at 670-700 nm showed the cumulative distribution of integrated DTX-VNS (Integrated VNS) over time. One hour postinjection, many DTX-VNS were located in the liver (red arrow). Its accumulation in the A549 tumor site increased gradually with time, and reached a maximum 48 hours after the injection. Interestingly, no strong fluorescent signal at 600-630 nm from 1-96 hours was observed in the liver, which meant that no NR or DiD was released from the nanosystem (Cracked VNS). However, this signal was clearly observed at the A549 tumor site at the 8th hour after injection (green arrow) and gradually enhanced from 8 to 48 hours, indicating obvious drug release behavior (Figure 8A, C). The nanosystem showed a similar drug release behavior in mice bearing 4T1 or MDA-MB-231 tumors (Figure S12). These results indicated the selective drug release behavior of DTX-VNS, with significantly faster drug release in tumors than in normal tissues, which may be another contributor to the lower systemic toxicity of DTX-VNS.

To investigate the mechanism of selective drug release, a NR- and DiD-loaded nanosystem without a VE component (DTX-NS) was prepared and injected into mice bearing A549 tumors. The difference in drug release rates in the tumors and normal tissues was obviously reduced compared to the DTX-VNS group (Figure 8B, D). As a possible explanation, more ROS in tumors than in normal tissues leads to the accelerated disintegration of VE-based nanosystems due to their antioxidant activity, which leads to a faster drug release rate. When VE is absent from the nanosystem, its disintegration will not be affected by ROS in tumors, and thus DTX-NS presents a nonselective drug release behavior in tumors and normal tissues.

### Biodistribution Study

To eliminate interference, we also investigated the distribution of DIR-VNS *in vivo*. The results showed that the DiR accumulation in the body increased with time, and accumulated substantially at the tumor sites (Figure S13).

The DTX distribution *in vivo* after the administration of DTX-VNS or Taxotere was further investigated. Figure 8E showed the concentration of DTX in the main organs including tumors 24 hours postinjection of DTX-VNS and Taxotere using a HPLC-MS assay. Compared with Taxotere, DTX-VNS resulted in significantly more DTX retention in organs, especially in tumors (over 50-fold higher DTX concentration). Higher DTX levels in tumors are another reason why DTX-VNS has stronger antitumor activity.

## Discussion

Docetaxel is one of the most widely used anticancer drugs. However, due to its poor solubility, absolute ethanol and Tween 80 are often adopted as dissolving mediums, which would frequently cause more serious side effects, such as neutropenia, hemolytic toxicity, myelosuppression, and hypersensitivity reaction 5, 16, 46. Since systemic toxicity limits DTX application in the clinic, we developed a VE-based strongly reductive nanosystem to increase the loading efficiency of docetaxel, reduce its side toxicity and enhance its antitumor effects.

We first investigated the *in vivo* antitumor activity of DTX-VNS. DTX-VNS exhibited significantly stronger tumor growth inhibition than Taxotere in the three cancer models (Figure 2). Although the VE nanosystem alone (Blank-VNS) did not exhibit significant antitumor activity, our data demonstrated that VE plays an important role in the synergistic antitumor effect of DTX-VNS. This synergistic effect dose not enhance the direct killing effect of DTX on cancer cells (Figure 3A, C), but mainly upregulating the antitumor immunity *in vivo*. VE and its derivatives have frequently been reported to have immune-modulatory effects and have shown antitumor activity by inducing apoptosis of cancer cells 47, 48, enhancing the Th1type immune response and hindering Th2 cytokine production 43, 44. Our results showed that VE nanosystems alone can indeed cause some changes in the microenvironment of tumors. Interestingly, the synchronous VE and DTX delivery system (DTX-VNS) demonstrated a significant enhancement of the anti-tumor immune microenvironment compared with DTX or VE treatment alone. DTX-VNS-treated tumors had significantly higher IFN-γ level and more infiltration of CD8+ T cells (Figure 4I, J). Decreased IL-4 and elevated IL-2 levels suggested an enhanced Th1 antitumor immune response and a reduced immunosuppressive microenvironment (Figure 4K, L). Compared with Taxotere, DTX-VNS showed a significant increase in the accumulation of DTX in tumors (Figure 8E), which may be another reason for its stronger antitumor effects, although DTX needs to undergo a drug release process in order to exert its activity. Interestingly, a faster drug release behavior of DTX-VNS was shown in tumors compared with normal tissues (Figure 8, S11, S12), which may be related to the fact that increased ROS levels in tumors contribute to the disintegration of the nanosystems. This selective drug release behavior of our nanosystem partially alleviates the problem of diminished therapeutic efficacy due to slow drug release.

ROS-associated oxidative stress involves the mechanism of toxicity and side effects caused by most chemotherapies 11, 12, 45. We designed the use of VE, with its antioxidant capacity to deplete the increased ROS in cells induced by chemotherapeutic agents, thus alleviating the excessive oxidative stress and reducing the damage to the cells induced by these agents. DTX-VNS significantly reduced ROS levels in tumor cells and inhibited the increase in ROS levels in normal cells stimulated by DTX (Figure 6C, D). In addition, ROS-responsive DTX-VNS in cancer cells can release a small amount of DTX (Figure 6A, B). In the evaluation of myelosuppression, DTX-VNS showed significantly lower inhibitory toxicity to bone marrow cell growth than Taxotere under the same concentration of DTX (Figure 5A, B and S4A-C). Our data also demonstrated that DTX-VNS has significantly reduced hemotoxicity compared to Taxotere (Figure 5C, 5D and S4D). DTX-VNS also exhibited low acute toxicity, even at injection doses of up to 64 mg/kg, by the analysis of hematology and clinical chemistry (Table S2, S3). An almost 2-fold higher dose tolerance of DTX was obtained for the mice under multiple intravenous injections of DTX-VNS compared with that of Taxotere. Importantly, during the treatments, the stiffness of the hind limbs caused by DTX-VNS was significantly less severe, which may suggest reduced neurotoxicity. These results indicate that our nanosystem may have significantly reduced systemic toxicity while maintaining its antitumor effects. The ability to alleviate ROS-mediated oxidative stress may be the main reason why DTX-VNS significantly reduces toxicity and side effects compared with Taxotere.

Understanding the fate of the nanomedicine and the drug release behavior *in vivo* is very important for its therapeutic efficacy and biosafety. We developed a FRET system to reveal the drug release behavior and fate of DTX-VNS in animals after an injection, and found a selective drug release characteristic of the nansystem in tumors and normal tissues (Figure 7, 8). After intravenous administration, DTX-VNS resulted in more DTX accumulation in normal tissues than Taxotere, although the slow drug release may alleviate the potential systemic toxicity due to cumulative increase in the drug.

Excessive or too little ROS could affect the growth of tumor cells. Although there are many studies on strategies to increase ROS to cytotoxic levels, this method inevitably induces systemic toxicity, similar to standard chemotherapy 49-51. To utilize cellular redox changes to develop safe and effective therapeutic strategies, it is necessary to experimentally describe specific redox signaling pathways that are characteristics of cancer cell growth and survival in the future.

## Conclusion

In this study, we designed a plenty of vitamin E-based reductive nanosystem (DTX-VNS) that reduced systemic toxicity and enhanced the anti-tumor effect of docetaxel. In addition, because the reductive nanosystem can simultaneously reduce the accumulation of intracellular reactive oxygen species (ROS) and alleviate cellular oxidative stress by the antioxidant effect of VE, it significantly reduced the side effects of myelosuppression, neurotoxicity and hemolysis. Compared to Taxotere, mice in the DTX-VNS group tolerated over a 2-fold higher total dose of DTX after multiple intravenous injections. Through FRET analysis, we found that our nanosystem showed a selective and more rapid release of drugs in tumors compared to normal organs due to the higher levels of ROS in tumor cells than normal cells, and the accumulation of DTX at tumor sites in the DTX-VNS group was also notably greater than that in the Taxotere group 24 hours after injection. Moreover, DTX-VNS had a prominently stronger anti-tumor effect in various models compared with Taxotere, and had a synergistic effect of immunotherapy. Our work presents a useful reference for the clinical exploration of the *in vivo* behavior of nanocarriers, inhibition of oxidative stress and selective release of drugs at tumor sites, thus reducing the side effects and enhancing the antitumor effects.

## Abbreviations

DTX: docetaxel
DTX-VNS: DTX-loaded vitamin E nanosystems
FRET: Förster Resonance Energy Transfer
ROS: reactive oxygen species
MCT: medium chain triglyceride
S100: soybean lecithin
VE: vitamin E
DMEM: Dulbecco's modified Eagle medium
RPMI: Roswell Park Memorial Institute
FBS: fetal bovine serum
MTT: 3-[4,5-Dimethylthiazol-2-yl]-2,5-diphenyltetrazolium bromide
DiO: 3,3′-dioctadecyloxacarbocyanine perchlorate
DiI: 1,1'-dioctadecyl-3,3,3',3'-tetramethylindocarbocyanine perchlorate
DiR: 1,1'-dioctadecyl-3,3,3',3'-tetramethylindotricarbocyan ine Iodide
DiD: propidium iodide
NR: Nile Red
H_2_O: distilled water
HPLC: high-performance liquid chromatography
HPH: high pressure homogenization
DTX-NS: DTX-loaded nanosystems, without VE
DLS: dynamic light scattering
TEM: transmission electron microscope
Blank VNS: blank nanosystem
