# Supplementary material for: Selective Intratumoral Drug Release and Simultaneous Inhibition of Oxidative Stress by a Highly Reductive Nanosystem and Its Application as an Anti-tumor Agent
Source: Theranostics. 2020 Jan 1;10(3):1166–80. doi: 10.7150/thno.38627 (PMC6956823; doi:10.7150/thno.38627)
Supplement: Supplementary file 1 — Supplementary figures and tables. [file thnov10p1166s1.pdf]

Supplementary Materials

Selective Intratumoral Drug Release and Simultaneous Inhibition of Oxidative Stress by a Highly Reductive Nanosystem and Its Application as an Anti-tumor Agent

*Chunqi Zhu[1], Lihua Luo[1], Xindong jiang[1], Mengshi Jiang[1], Zhenyu Luo[1], Xiang Li[1], Weigen Qiu[1], Zhaolei Jin[1], Tianxiang Shen[1], Chunlong Li[1], Qingpo Li[1], Yunqing Qiu[2] and Jian You[1]**

Authors' Affiliations:

[1]College of Pharmaceutical Sciences, Zhejiang University, 866 Yuhangtang Road, Hangzhou, Zhejiang 310058, P. R. China

[2]State Key Laboratory for Diagnosis and Treatment of Infectious Diseases, Collaborative Innovation Center for Diagnosis and Treatment of Infectious Diseases, The First Affiliated Hospital, Zhejiang University, 79 Qingchun Road, Hangzhou, Zhejiang 31003, P. R. China

* Corresponding Author:

Jian You, College of Pharmaceutical Sciences, Zhejiang University, 866 Yuhangtang Road, Hangzhou 310058, Zhejiang, China. Office: 086-571-88981651, Email: youjiandoc@zju.edu.cn.

Table S1. Size, Polydispersity Index (PDI), Zeta Potential and the content of DTX of the DTX-VNS.

Days	4 ℃				25 ℃			
	d_n (nm)	PDI	ζ (mV)	C (mg/g)	d_n (nm)	PDI	ζ (mV)	C (mg/g)
1	108.5 ± 3.4	0.199	-37.6 ± 2.3	2.10	108.5 ± 3.4	0.199	-37.6 ± 2.3	2.10
7	109.5 ± 2.6	0.172	-37.3 ± 1.2	2.06	114.0 ± 2.7	0.134	-39.7 ± 1.4	2.05
14	109.5 ± 1.3	0.161	-32.9 ± 2.6	2.04	115.4 ± 0.7	0.108	-34.5 ± 1.4	2.03
30	119.9 ± 1.4	0.147	-34.1± 1.3	2.02	118.9 ± 2.0	0.130	-36.3 ± 1.2	2.00
60	113.5 ± 2.3	0.161	-40.5± 1.5	2.05	124.4 ± 4.3	0.142	-39.2 ± 0.9	2.03

The dn, PDI, ζ and C values represent the diameter, PDI, zeta potential and the content of DTX in DTX-VNS, respectively.

Table S2 The mean and standard deviation (SD) values for each of the hematologic variables.

Groups	WBC (10^9/L)	%NEUT (%)	%LYMPH (%)	%MONO (%)	%EO (%)	%BASO (%)	RBC (10^{12}/L)	Hb (g/dL)	Hct (%)	MCV (fL)	MCH (Pg)	MCHC (g/dL)	PLT (10^9/L)	RET (10^9/L)
Saline	3.87±1.06	13.8±5.1	83.0±3.5	2.2±1.3	1.0±0.4	0.0±0.0	6.83±0.31	13.2±0.4	37.3±1.2	54.6±0.9	19.3±0.4	35.4±0.3	859±51	232.2±51.2
Blank VNS	7.22±2.77	26.9±22.1	68.1±19.4	3.9±2.6	1.1±0.3	0.0±0.0	6.67±0.28	12.8±0.4	37.1±1.1	55.6±1.9	19.2±0.4	34.6±0.4	866±112	365.1±63.6
DTX-VNS N	1.90±1.87	23.2±1.0	70.7±2.0	5.2±1.6	0.0±0.0	0.9±0.6	5.16±0.67	10.7±0.9	32.3±2.1	63.0±3.9	20.8±1.0	33.1±0.6	683±135	934.7±33.5
DTX-VNS H	2.61±0.88	23.3±14.0	72.3±14.7	4.2±0.8	0.0±0.0	0.2±0.3	5.36±0.12	10.9±0.3	32.8±1.4	61.1±2.1	20.3±0.5	33.2±0.5	732±150	1,092.7±306.7
Taxotere N	3.49±0.54	8.6±4.3	83.1±2.4	7.8±3.0	0.0±0.0	0.5±0.3	4.79±0.67	9.8±1.3	29.7±3.4	62.2±2.5	20.5±0.7	33.0±0.7	554±143	729.7±236.4
Taxotere H	1.92±0.55	10.4±3.5	80.7±6.8	8.3±3.3	0.0±0.0	0.6±0.2	5.16±0.74	10.0±1.4	29.5±4.2	57.1±0.9	19.4±0.2	34.0±0.4	358±141	467.4±145.3

Table S3 The mean and standard deviation (SD) values for each of the blood biochemical variables.

Groups	TP	Alb	ALT	AST	TB	ALP	BUN	Cr	Glu	K	Na	Cl	TG	TC	CK
	g/L	g/L	U/L	U/L	μmol/L	U/L	mmol/L	μmol/L	mmol/L	mmol/L	mmol/L	umol/L	mmol/L	mmol/L	U/L
Saline	59.5±3.2	46.5±2.2	21.9±2.2	77.3±6.4	1.0±0.2	59±17	4.6±0.4	29±4	6.39±0.53	3.66±0.12	140±1	101.1±0.8	0.25±0.03	1.59±0.22	389±55
Blank VNS	61.3±2.5	45.0±3.2	75.9±76.2	147.7±62.1	1.3±0.1	98±9	5.8±1.6	35±8	6.57±0.17	3.65±0.26	140±1	100.3±1.3	0.22±0.11	0.83±0.07	390±161
DTX-VNS N	48.0±3.0	38.4±3.9	25.7±4.3	109.5±4.0	1.1±0.4	46±18	6.5±0.3	22±1	6.21±0.25	4.13±0.17	140±2	100.0±0.5	1.00±0.55	1.73±0.41	332±83
DTX-VNS H	49.7±2.4	36.8±0.5	50.6±11.6	146.7±27.6	1.2±0.2	41±4	8.3±1.9	24±3	7.00±1.45	4.09±0.15	140±1	97.8±2.2	0.29±0.12	2.48±0.57	341±43
Taxotere N	45.7±5.0	35.5±4.0	29.5±1.2	99.4±14.9	1.9±0.2	37±7	8.8±0.7	27±4	6.91±0.43	4.19±0.20	141±1	100.6±1.0	0.71±0.26	2.16±0.26	222±34
Taxotere H	50.2±3.9	35.5±3.8	28.6±3.0	134.0±31.2	2.0±0.6	59±28	10.0±1.9	23±6	6.81±1.05	4.30±0.36	140±2	101.4±1.4	0.33±0.11	2.23±0.30	289±66

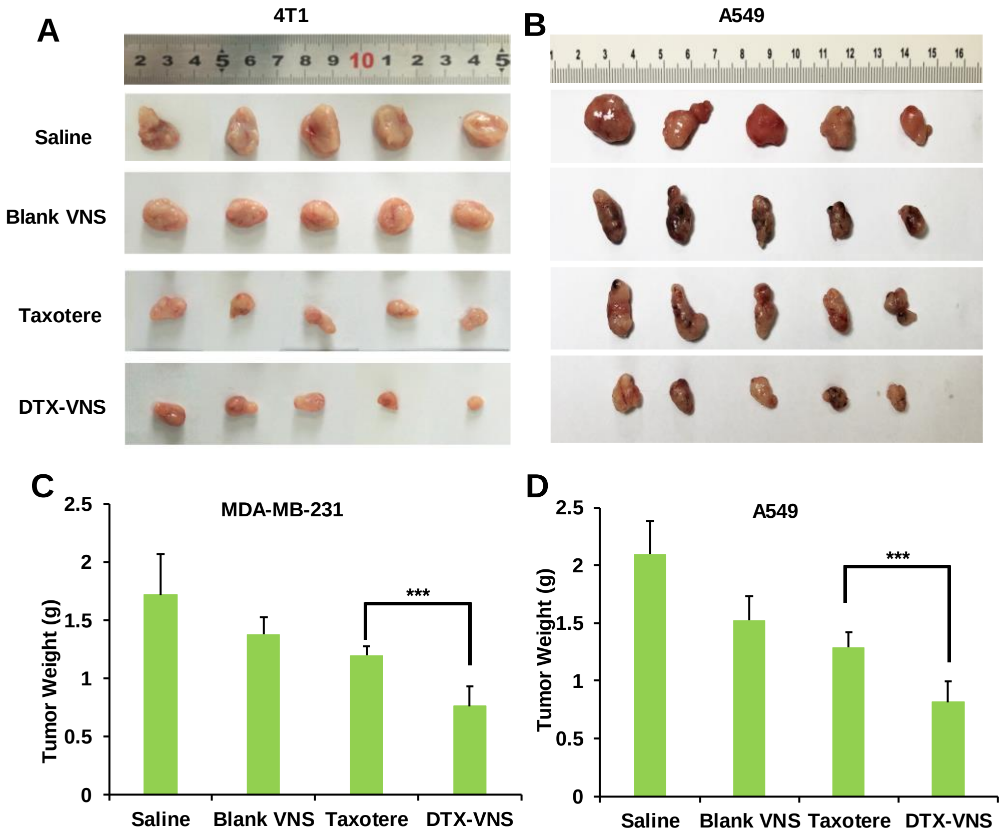

Figure S1 Representative tumor imaging of each group of 4T1 (**A**) and A549 (**B**) tumor models at the end of the study. Average tumor weight of each group of MDA-MB-231 (**C**) and A549 (**D**) tumor models at the end of the study. *p < 0.05, **p < 0.01, ***p <0.005.

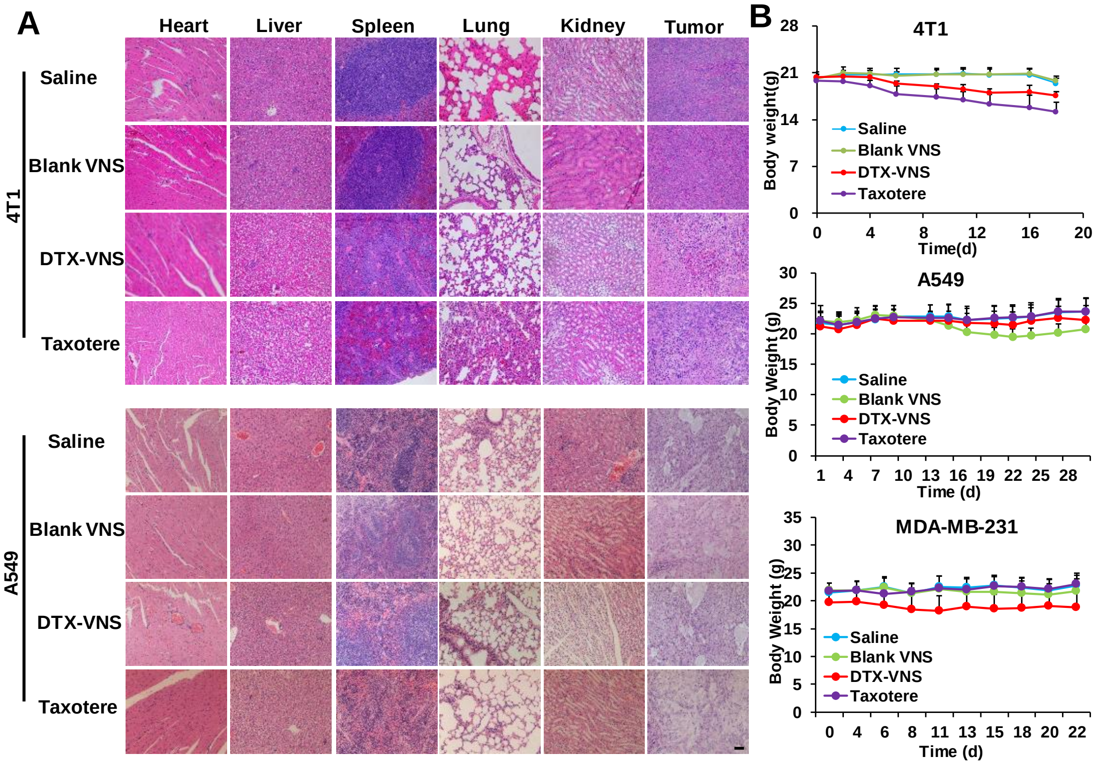

Figure S2 *In vivo* anticancer effect for the mice bearing different tumors. (**A**) Representative H&E staining images of various tissues (heart, liver, spleen, lung, kidney, and tumor) for four groups; the scale bar is 100 µm. (**B**) The mean body weight in the whole experiment process (n=5).

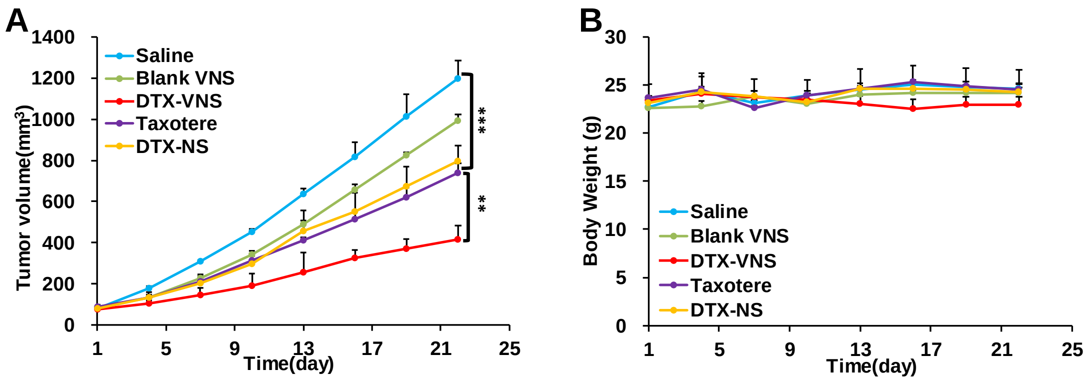

Figure S3 *In vivo* anticancer effect for the mice bearing 4T1 tumors. (**A**) Tumors growth curves. (**B**) The mean body weight in the whole experiment process. *p < 0.05, **p < 0.01, ***p <0.005.

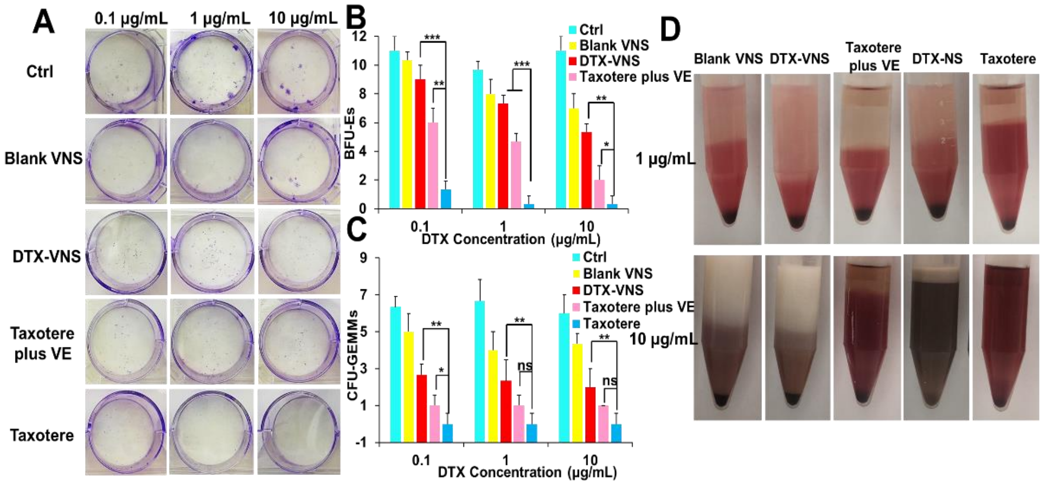

Figure S4 The detoxification of DTX-VNS. (**A**) Bone marrow cells from mice were incubated with Blank VNS, DTX-VNS, Taxotere, and Taxotere plus VE at the dose of 0.1 μg/mL, 1 μg/mL or 10 μg/mL for 14 days in methylcellulose-based media. Burst-forming units that generate erythroids (**B**) and colony forming units that generate granulocytes, erythroids, macrophages, and megakaryocytes (**C**) were counted using an inverted microscope. The data are presented as mean ± standard error from three experiments. *p < 0.05, **p < 0.01, ***p <0.005. (**D**) Photographs of defibrinated blood mixed with with Blank VNS, DTX-VNS, Taxotere plus VE, DTX-NS, and Taxotere in different concentration (high: 10 μg/mL; low: 1 μg/mL) for 3 hours.

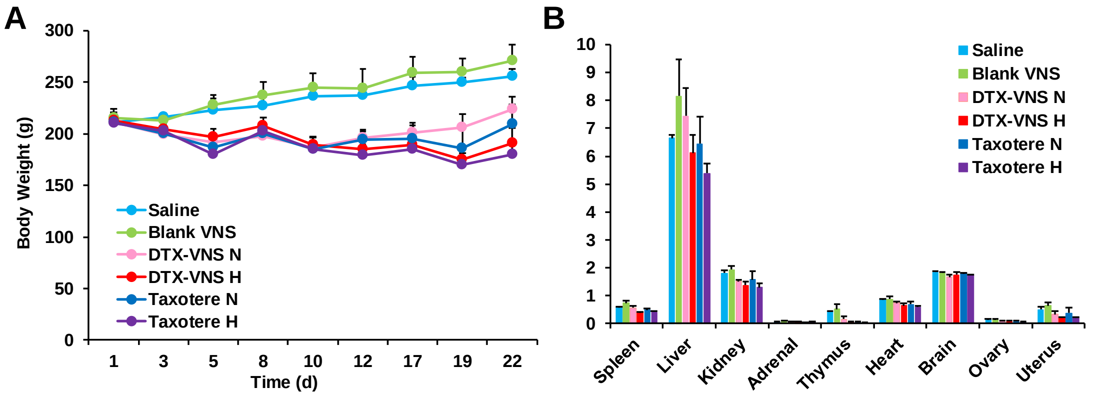

Figure S5 Toxicity evaluation. (**A**) Rats body weight changes under different treatment in the whole experiment process. (**B**) Relative organ weights of rats receiving different treatments for 22 days.

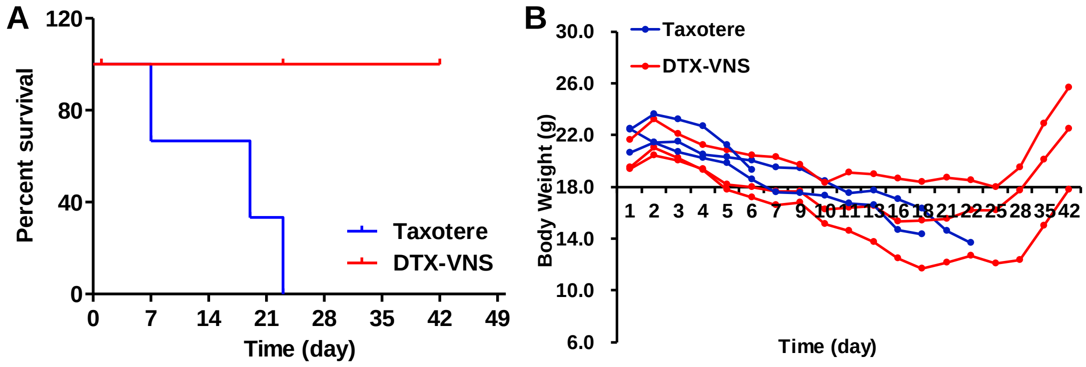

Figure S6 The tolerance dose study. (**A**) The percent survival of the mice after treated with Taxotere and DTX-VNS. (**B**) Individual body weight of the mice in the whole experiment process (n=3).

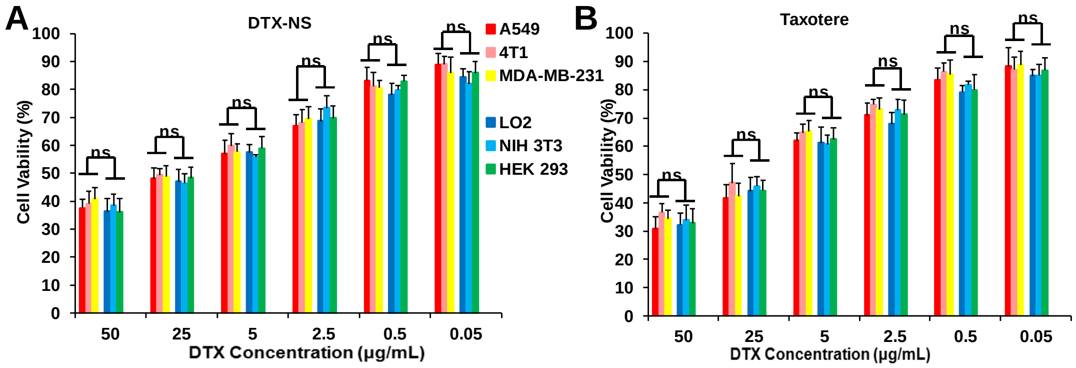

Figure S7 MTT assay of three tumor cells (A549, 4T1, MDA-MB-231) and three normal cells (LO2, NIH 3T3, HEK 293) treated with DTX-NS (**A**) or Taxotere (**B**) for 48 hours (n=5). All error bars are expressed as ± SD, n=5; *p < 0.05, **p < 0.01, ***p <0.005.

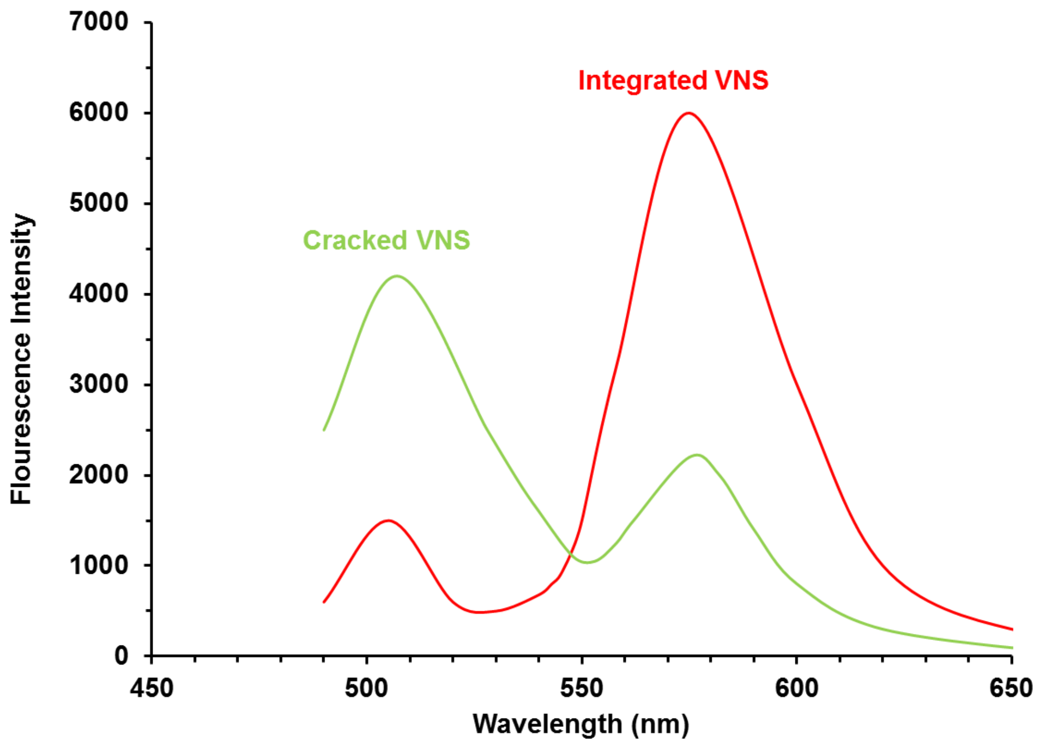

Figure S8 Spectra of DiO-, DiI-loaded DTX-VNS diluted by 100 X water (red curve, represented Integrated VNS) and 100 X alcohol (green curve, represented Cracked VNS), respectively.

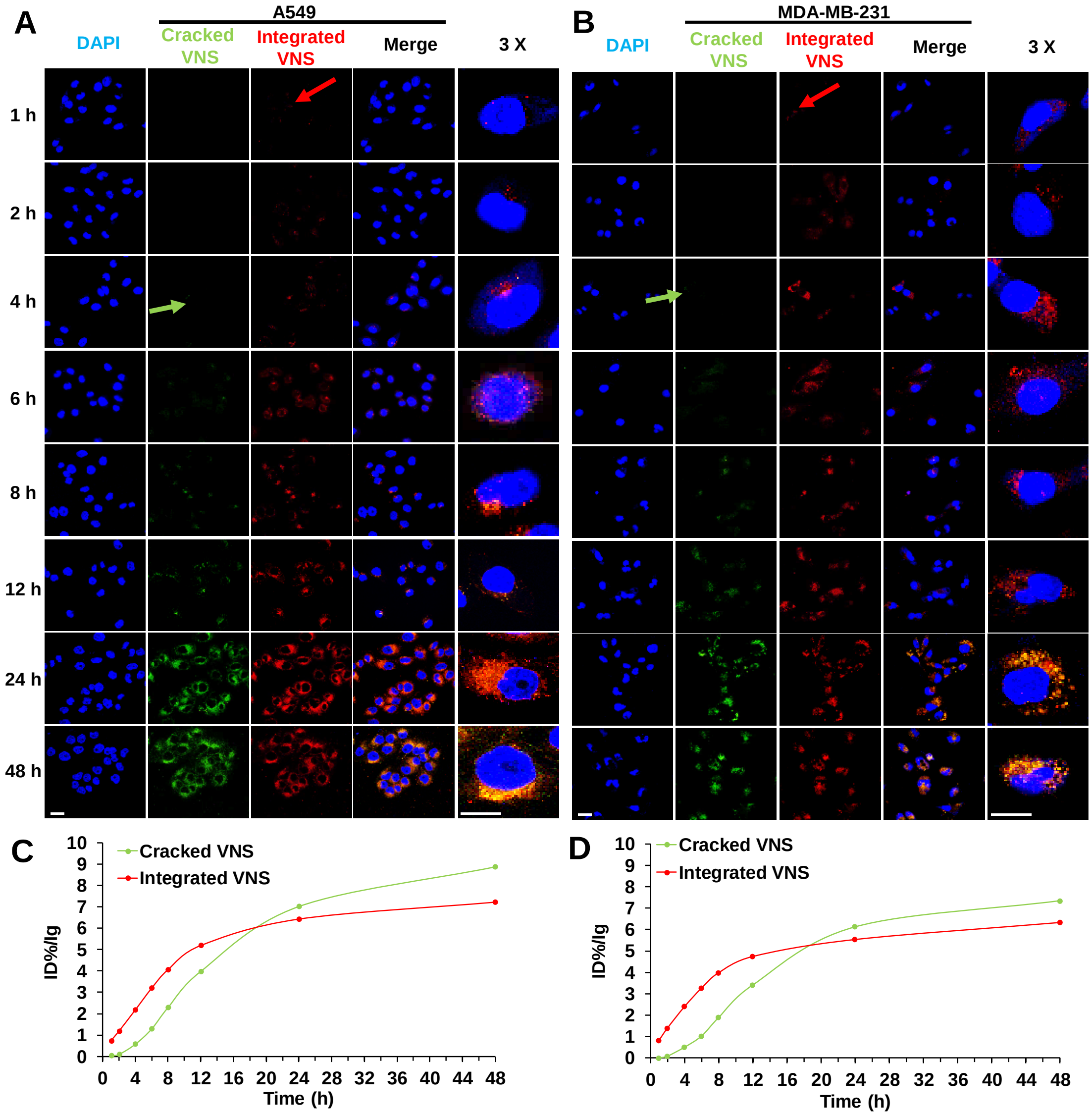

Figure S9 *S*elective release of DTX-VNS in tumor cells. Cellular uptake of DiO-, DiI-loaded DTX-VNS were incubated with A549 (**A**) and MDA-MB-231 (**B**) tumor cells at 37 °C for predetermined times (blue: cell nuclei; green: cracked VNS; red: integrated VNS), and the fluorescence images inspected by confocal fluorescence microscopy. The fluorescence intensity of cracked VNS and integrated VNS in A549 (**C**) or MDA-MB-231 tumor cells (**D**) was calculated based on (**A**) or (**B**) using ImageJ; the scale bar is 20 μm.

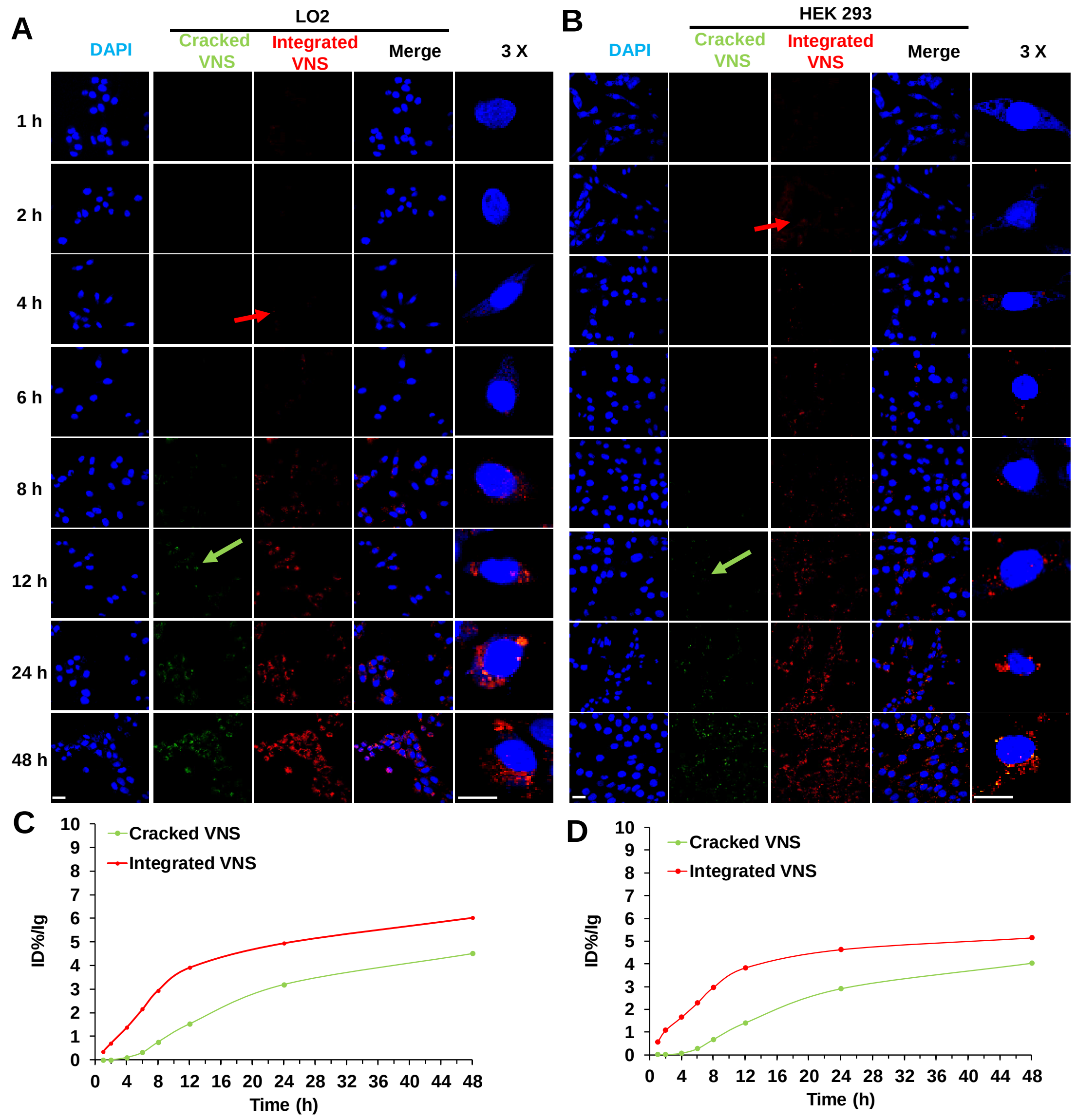

Figure S10 *S*elective release of DTX-VNS in normal cells. Celluar uptake of DiO-, DiI-loaded DTX-VNS were incubated with LO2 (**A**) and HEK 293 normal cells (**B**) at 37 °C for predetermined times (blue: cell nuclei; green: Cracked VNS; red: Integrated VNS), and the fluorescence images inspected by confocal fluorescence microscopy. The fluorescence intensity of Cracked VNS and Integrated VNS in LO2 (**C**) or HEK 293 normal cells (**D**) was calculated based on (**A**) or (**B**) using ImageJ; the scale bar is 20 μm.

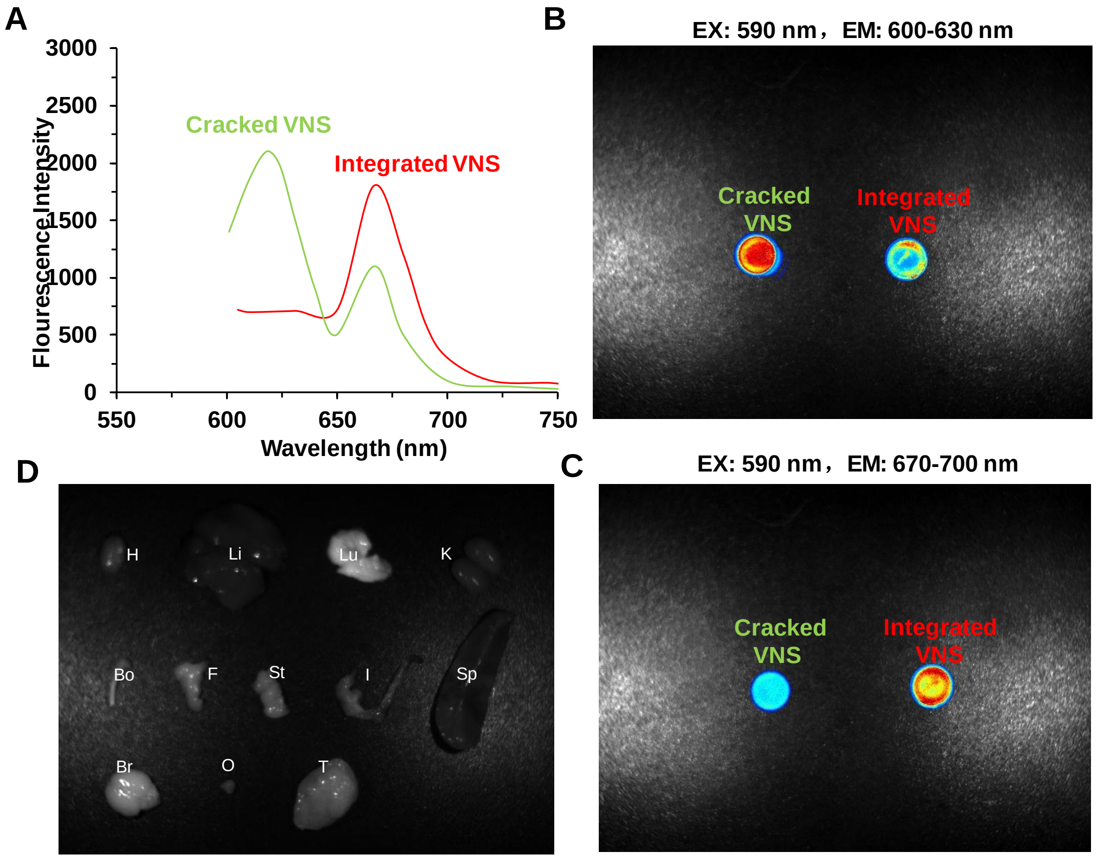

Figure S11 *In vitro* selective release of DTX-VNS. (**A**) Spectra of NR-, DiD-loaded DTX-VNS diluted by 100 X water (red curve, represented Integrated VNS) and 100 X alcohol (green curve, represented Cracked VNS), respectively. Fluorescence imaging of NR-, DiD-loaded DTX-VNS diluted by and 100 X alcohol (left, represented Cracked VNS) and 100 X water (right, represented Integrated VNS), respectively. The excitation was 590 nm, and the emission were 600-630 nm (**B**) or 670-700 nm (**C**). (**D**) Optical photos of various tissues (H: heart, Li: liver, Lu: lung, K: kidney, Bo: bone, F: fat, St: stomach, I: intestines, Sp: spleen, Br: brain, O: ovary, and T: tumor).

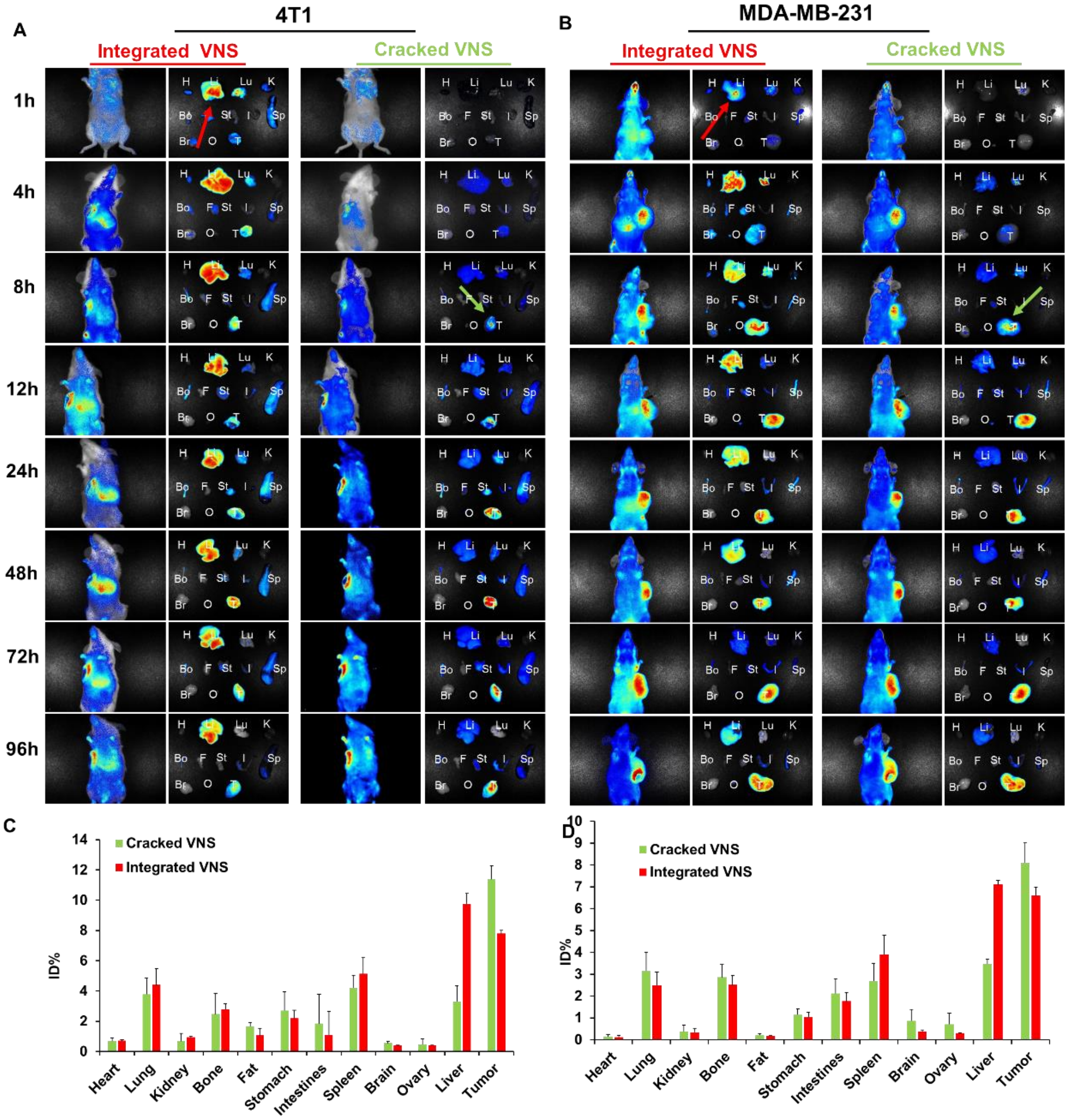

Figure S12 *In vivo* and ex *vivo* selective release of DTX-VNS. *In vivo* and *ex vivo* images of the mice bearing 4T1 tumors and various tissues (**A**) or MDA-MB-231 tumors and various tissues (**B**) were acquired at predetermined times post i.v. injection. %ID/g (percentage of the injected dose per gram of tissue) was analyzed to show the accumulation of cracked VNS and integrated VNS in 4T1 tumors and various tissues (**C**) or MDA-MB-231 tumors and various tissues (**D**) at 24 h after i.v. injection.

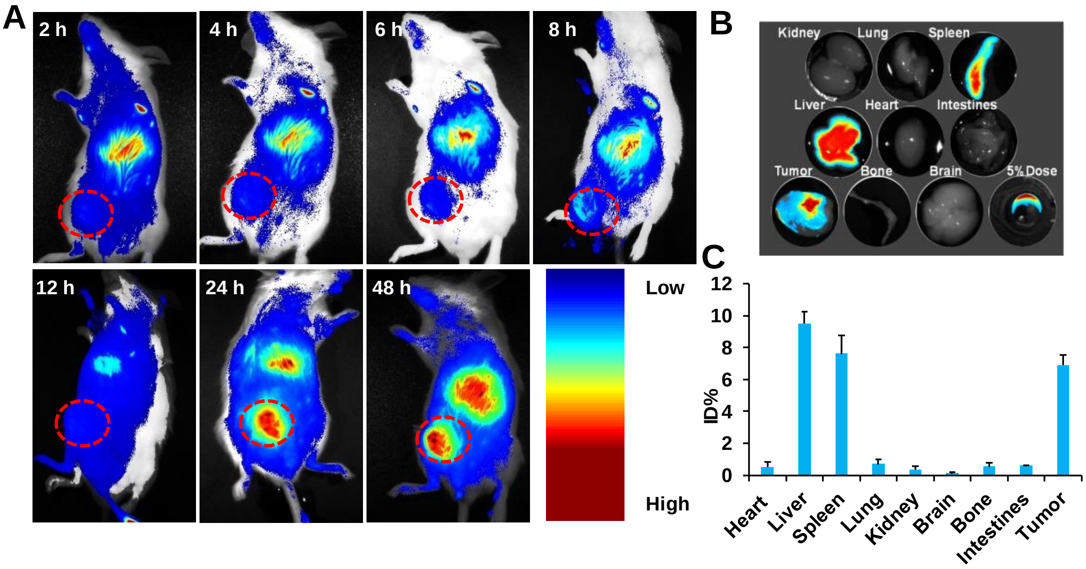

Figure S13 (**A**) *In vivo* fluorescence imaging of 4T1 tumor bearing mince i.v. injected with DiR-labeled DTX-VNS at different time points. (**B**) Fluorescent images of various tissues distribution of DTX-VeNP at 48 hours after injection. (**C**) Quantitative analysis of *ex vivo* fluorescence images in (**B**).
